# Proteomic analysis of fibroblastema formation in regenerating hind limbs of *Xenopus laevis* froglets and comparison to axolotl

**DOI:** 10.1186/1471-213X-14-32

**Published:** 2014-07-25

**Authors:** Nandini Rao, Fengyu Song, Deepali Jhamb, Mu Wang, Derek J Milner, Nathaniel M Price, Teri L Belecky-Adams, Mathew J Palakal, Jo Ann Cameron, Bingbing Li, Xiaoping Chen, David L Stocum

**Affiliations:** 1 Department of Biochemistry and Genetics, School of Medicine, American University of Antigua, Coolidge, Antigua, West Indies; 2 Department of Oral Biology, School of Dentistry and Center for Developmental and Regenerative Biology, Indiana University-Purdue University Indianapolis, Indianapolis, IN, USA; 3 School of Informatics and Computing, Indiana University-Purdue University Indianapolis, Indianapolis, IN, USA; 4 Department of Biology, Indiana University-Purdue University Indianapolis, Indianapolis, IN, USA; 5 Department of Biochemistry and Molecular Biology, School of Medicine, and Center for Developmental and Regenerative Biology, Indiana University-Purdue University Indianapolis, Indianapolis, IN, USA; 6 Department of Biology, and Center for Developmental and Regenerative Biology, Indiana University-Purdue University Indianapolis, Indianapolis, IN, USA; 7 Department of Chemistry, Central Michigan University, Mt. Pleasant, MI, USA; 8 School of Informatics and Computing, and Center for Developmental and Regenerative Biology, Indiana University-Purdue University, Indianapolis, IN, USA; 9 Department of Cell and Developmental Biology, and Regeneration Biology and Tissue Engineering Theme, Institute for Genomic Biology, University of Illinois-Urbana Champaign, Urbana, IL, USA

**Keywords:** Regeneration, *Xenopus* hindlimb, Proteomic analysis, Fibroblastema formation, Comparison to axolotl

## Abstract

**Background:**

To gain insight into what differences might restrict the capacity for limb regeneration in *Xenopus* froglets, we used High Performance Liquid Chromatography (HPLC)/double mass spectrometry to characterize protein expression during fibroblastema formation in the amputated froglet hindlimb, and compared the results to those obtained previously for blastema formation in the axolotl limb.

**Results:**

Comparison of the *Xenopus* fibroblastema and axolotl blastema revealed several similarities and significant differences in proteomic profiles. The most significant similarity was the strong parallel down regulation of muscle proteins and enzymes involved in carbohydrate metabolism. Regenerating *Xenopus* limbs differed significantly from axolotl regenerating limbs in several ways: deficiency in the inositol phosphate/diacylglycerol signaling pathway, down regulation of Wnt signaling, up regulation of extracellular matrix (ECM) proteins and proteins involved in chondrocyte differentiation, lack of expression of a key cell cycle protein, ecotropic viral integration site 5 (EVI5), that blocks mitosis in the axolotl, and the expression of several patterning proteins not seen in the axolotl that may dorsalize the fibroblastema.

**Conclusions:**

We have characterized global protein expression during fibroblastema formation after amputation of the *Xenopus* froglet hindlimb and identified several differences that lead to signaling deficiency, failure to retard mitosis, premature chondrocyte differentiation, and failure of dorsoventral axial asymmetry. These differences point to possible interventions to improve blastema formation and pattern formation in the froglet limb.

## Background

Urodeles regenerate perfect replicas of limb segments lost by amputation at any proximodistal (PD) level throughout their lives [1,2], for reviews, although the rate and completeness of regeneration are affected by factors such as age and metamorphosis [3]. Regeneration is accomplished by the formation of a blastema composed of progenitor cells derived by reprogramming of differentiated cells (dedifferentiation) and stem cells associated with skeletal muscle and perhaps other tissues. Growth of the blastema is driven by a nerve-dependent signaling center, the apical epidermal cap (AEC) [4-8]. Global transcript analysis by microarray and RNA-Seq has identified overlapping suites of genes that include markers for stem and progenitor cells, genes that define specific phases of regeneration, genes that are regulated by neural signals, and genes that differentiate regeneration from skin wound repair [9-11].

Nieuwkoop-Faber [12] stage 51–53 limb buds of the anuran *Xenopus laevis* also regenerate perfectly at any level of amputation. After NF stage 53, however, regenerative capacity becomes progressively hypomorphic and spatially restricted to progressively more distal levels, until by stage 56 or 57 amputation at any level results only in the regeneration of a muscle-less, un-segmented cartilage spike covered by an envelope of skin [13-15]. This spatiotemporal restriction of regenerative capacity is correlated with the general proximal to distal ossification of skeletal tissues, although regeneration is slightly better when amputation is through the soft tissue of the joints [16]. Loss of regenerative capacity during limb development in *Xenopus* is due to intrinsic changes in limb tissues, as shown by the fact that grafting regeneration-competent blastemas to regeneration-deficient limb stumps and *vice versa* does not alter the regenerative capacity of the blastema [17,18].

The *Xenopus* and urodele limb regeneration blastema share some features. Both rely on nerve-dependent signals from the wound epidermis for their formation and growth [19-22]. Both express *prx1*, a transcription factor that serves as an early marker of dedifferentiated cells [23,24]. Most often, however, the *Xenopus* blastema is described as a “fibroblastema” or “pseudoblastema”, as opposed to the mesenchymal nature of the urodele blastema. Although one study [25] reported that the morphology and fine structure of the cells released by histolysis is similar in amputated urodele and *Xenopus* limbs, most studies suggest that, compared to the amputated urodele limb, histolysis is limited in the amputated *Xenopus* limb, there is little if any cellular dedifferentiation, progenitor cells are fibroblastic rather than mesenchymal, muscle satellite cells do not contribute to the fibroblastema, neurovascular invasion is sparser, and the AEC is thinner with a connective tissue pad between it and the underlying cells [13,16,20,26,27]. These features have been correlated with a shift in the response to amputation brought about by the maturation of the immune system as the tadpole differentiates and undergoes metamorphosis [28-30].

Defining the cellular and molecular basis of the contrast in regenerative ability between regeneration-competent and regeneration-deficient limbs is of great interest, because of the potential to identify factors associated with successful regeneration and/or the factors that inhibit it. Differences in transcript expression by amputated regeneration-competent *Xenopus* limb buds (stage 52/53) vs. regeneration-deficient limbs (stage 57 or froglets) have been reported for specific genes and for global gene arrays compiled by subtractive hybridization or microarray [31-34]. In particular, PD axial patterning genes such as *Hoxa9, Hoxa11,* and *Hoxa13* are expressed by the fibroblastemas of *Xenopus* limbs, but their expression is not deployed in the proper spatiotemporal organization characteristic of regeneration-competent blastemas [35]. Furthermore, regeneration-deficient *Xenopus* blastemas fail to express *shh*, an important regulator of anteroposterior (AP) axial patterning in axolotl limb buds and blastemas and *Xenopus* stage 52 limb buds [31], a failure due to the epigenetic hyper-methylation of an enhancer sequence regulating *shh* expression [36]. These findings have led to the idea that faulty expression of patterning genes is the major cause of regenerative deficiency in *Xenopus* limbs [35]. The reasons why *Xenopus* limb patterning genes are not activated in their proper spatiotemporal pattern are unknown, but are likely due to an inability to activate and/or inhibit other processes necessary to the formation of a regeneration-competent blastema.

Analysis of gene activity on the mRNA level excludes post-transcriptional events that determine whether or not specific transcripts are translated into protein and at what rate. Transcript analysis has therefore been complemented by proteomic analysis of regenerating amphibian limbs. Protein synthesis in regenerating newt limbs has been studied by autoradiographic [37], biochemical [38,39] and gel electrophoretic [40-43] methods. The electrophoretic studies revealed differences in protein composition at different stages of regeneration and between innervated and denervated limbs, but only a few proteins were identifiable. Quantitative HPLC/mass spec methods have enabled the identification of individual proteins and their fold changes with respect to baseline values. King et al. [44] compared the blastemal proteome of stage 53 *Xenopus* limb buds at three days post-amputation to un-amputated tissue and identified a number of proteins with large fold changes. We have assessed temporal quantitative changes in proteins expressed during formation of the accumulation (early bud) blastema of the regenerating axolotl hind limb [45], and have conducted bioinformatic analysis to reveal pathways and networks of protein interactions during blastema formation [46].

To gain insight into the proteomic differences between blastema formation in regeneration-deficient *Xenopus* and regeneration-competent axolotl limbs, we have conducted a proteomic analysis of fibroblastema formation in *Xenopus* froglets and compared the results to those we obtained for blastema formation in the axolotl limb [45]. This species comparison has advantages over comparisons between regeneration-competent stage 52/53 *Xenopus* tadpole limb buds vs. regeneration-deficient stage 57-60 tadpole or froglet limbs in that we are comparing the regeneration of fully differentiated, nerve-dependent limbs rather than undifferentiated nerve-independent limb buds vs. nerve-dependent differentiated limbs.

## Results and discussion

### Histology of fibroblastema formation in froglet hind limbs

**Figure 1:**
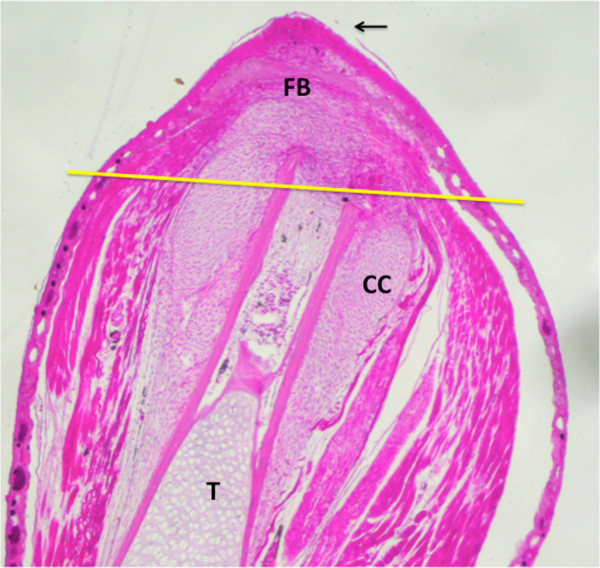
**Histological section of regenerating froglet hindlimb at 12 days post-amputation through the mid tibia-fibula.** The section is cut through the dorsoventral plane such that the tibia (T) and the accompanying flexor and extensor muscle masses are visible (red). A cartilage collar (CC) has formed around the tibia for some distance proximal to the amputation plane that merges distally with a similar collar surrounding the fibula. A fibroblastema (FB) that will form the cartilage spike is present between the merge point of the cartilage collars and the apical epidermal cap (arrow). Yellow line indicates the plane of tissue harvest.

At 1 dpa, the wound epidermis covered the clotted plasma of the amputation surface with its blood cells and cellular and tissue debris. By 5 dpa, the debris had been largely removed by macrophages and tissue histolysis by proteolytic degradation was underway. By 7 dpa, cells in the periosteum were activated for a considerable distance proximal to the amputation plane, forming two thick collars of fibroblastic cells surrounding both tibia and fibula that merged distally into a thin region of fibroblastic cells under the wound epidermis. By 12 dpa (Figure 1), the collar was differentiating into chondrocytes that graded into a mound of fibroblastic cells representing an accumulation fibroblastema. The fibroblastema grows as it simultaneously differentiates into chondrocytes in continuity with the cartilage collar to form the cartilage spike that is the characteristic end point of the regenerating froglet limb.

### *Xenopus* protein expression

**Figure 2:**
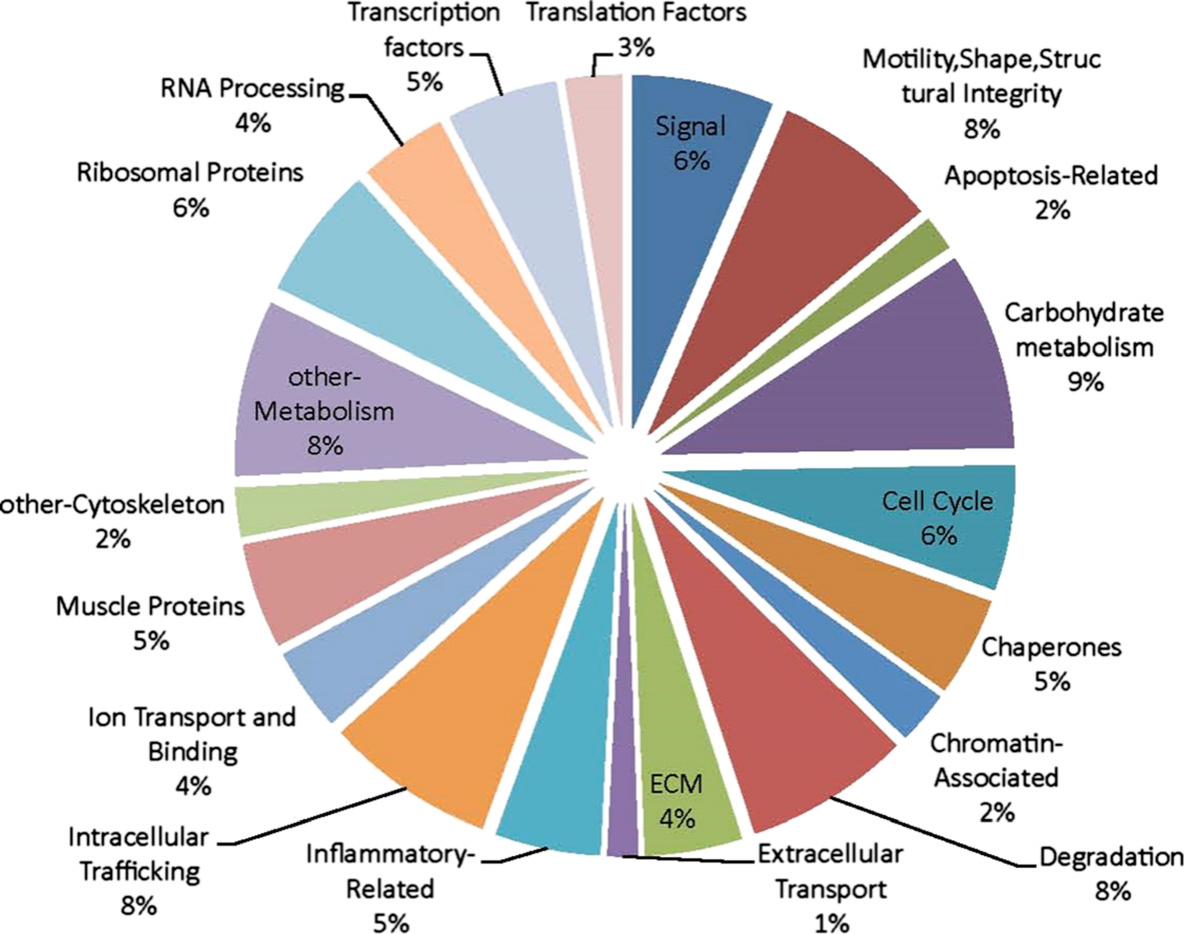
Pie chart showing the percentage distribution of the 820 proteins among different biological categories and sub-categories.

A total of 2500 Xenopus peptides were separated in the samples. They fell into four priority groups according to confidence in identification of proteins using a human database. Priority groups 1 (P1, 601 proteins) and 2 (P2, 613 proteins met a statistical cutoff of >90% (see Methods for further details). These 1214 proteins were filtered as outlined in Methods to give 1014 identifiable proteins. Collapsing duplicates and discarding proteins with no known function yielded 830 proteins for analysis. The remaining 1296 proteins fell into P3 and P4 categories that did not meet the cutoff and were therefore not included in the analysis. Figure 2 summarizes the distribution of P1/P2 proteins according to their biological process and Additional file 1: Table S1 lists all the P1/P2 proteins in each of the ten biological process categories/sub-categories, with fold change (FC) at each of the time points post-amputation, and peptide sequence. Proteins that had FC =/>2.0 are coded in yellow. Additional file 2: Figure S1 illustrates global intensity maps of FC at 1, 5, 7 and 12 dpa for each category of biological process.

Eighty percent of the P1 and P2 *Xenopus* proteins were up or down regulated at all post-amputation time points or three of four time points in the combinatorial expression patterns summarized in Table 1. The number and percentage of proteins in each biological process category up or down regulated at either all four or three of four time points, and the ratios of up to down regulation, are summarized in Table 2. The overall ratio of up regulated to down regulated proteins was 1.3. Up regulation was heavily favored over down regulation in the categories of signaling, transcription, translation, non-muscle cytoskeleton, ECM, and cell protection (particularly chaperones). Categories in which up regulation was moderately or slightly favored over down regulation were degradation and cell cycle. Down regulation was heavily favored in three categories, intracellular transport, cytoskeleton (particularly sarcomeric proteins), and metabolism (particularly carbohydrate metabolism).

**Table 1:** Patterns of up or down regulation observed at all four post-amputation time points or three of the four time points

**Up regulation**				**Down regulation**			
**1dpa**	**5 dpa**	**7 dpa**	**12 dpa**	**1 dpa**	**5 dpa**	**7 dpa**	**12 dpa**
u	u	u	u	d	d	d	d
d/n	u	u	u	u/n	d	d	d
u	u	u	d/n	d	d	d	u/n
u	d/n	u	u	d	u/n	d	d
u	u	d/n	u	d	d	u/n	d

u = up regulation, d = down regulation, n = no change. Slash means “or”; dpa = days post-amputation.

**Table 2:** Number of Xenopus proteins in each biological process category that are up regulated (U) or down regulated (D) on all dpa or 3 of 4 dpa, and U/D ratios

**Biological process**	**Total**			
**Category**	**Proteins**	**U**	**D**	**U/D**
Signaling	93	49 (52.7)	31 (33.3)	1.6
Transport	67	30 (46.6)	32 (50.0)	0.9
VT	44	20 (45.5)	24 (54.5)	0.8
NVT	23	10 (43.5)	8 (34.8)	1.3
Transcription	104	51 (49.0)	36 (34.6)	1.4
Chromatin associated	24	13 (54.2)	7 (29.2)	1.9
Transcription factors	41	19 (46.3)	16 (39.0)	1.2
RNA processing	39	19 (48.7)	13 (33.3)	1.5
Translation	78	48 (62.8)	15 (20.6)	3.1
Ribosomal proteins	55	34 (61.8)	10 (18.2)	3.4
Translation factors	23	15 (60.9)	5 (21.7)	3.0
Cytoskeleton	104	40 (38.5)	41 (39.4)	0.9
Muscle	28	3 (10.7)	23 (82.1)	0.1
Non-Muscle	76	37 (53.0)	18 (21.0)	2.1
Extracellular matrix	41	25 (61.0)	4 (9.8)	6.3
Metabolism	135	39 (37.1)	68 (50.3)	0.7
Carbohydrate	72	16 (22.2)	39 (54.2	0.4
Non-Carbohydrate	63	23 (36.5)	29 (44.4)	0.8
Cell Protection	80	39 (48.70)	21 (26.3)	1.9
Inflamm-Related	38	16 (42.1)	11 (28.9)	1.5
Apoptosis-Related	10	5 (50.0)	5 (50.0)	1.0
Chaperones	32	18 (56.3)	5 (15.6)	3.6
Degradation	64	27 (42.2)	21 (32.8)	1.3
Ubiquit/Proteasome	29	11 (37.9)	10 (34.4)	1.1
Other	35	16 (45.7)	11 (31.4)	1.5
Cell cycle	54	21 (38.9)	18 (33.3)	1.2
G1/S	15	6 (40.0)	7 (46.7)	0.9
M	18	8 (44.4)	6 (33.4)	1.3
Other	21	7 (33.3)	5 (23.8)	1.4
Total		369	287	1.3

Numbers in parentheses indicate percentages of the total proteins up or down regulated. VT = vesicle-associated transport and NVT non-vesicle-associated transport.

Two hundred seventy-five proteins (33.6% of the total) had positive or negative FC =/>2 on one or more dpa. Table 3 summarizes the ratio of up regulation to down regulation, in the patterns shown in Table 1, for these proteins in each biological process category, and Additional file 3 lists the designations of these proteins. The U/D ratio for these high FC proteins was highest (2–4) for signaling, translation, ECM and non-muscle cytoskeleton; intermediate (1–2) for transcription, cell protection, non-carbohydrate metabolism, degradation, and cell cycle; and lowest (<1) for intracellular transport, muscle cytoskeleton, and carbohydrate metabolism. Within the cytoskeleton category, U/D for muscle proteins was only 0.1 compared to non-muscle proteins at 3.7, and within the metabolism category it was 0.3 for carbohydrates in contrast to 1.3 for non-carbohydrate.

**Table 3:** Number of Xenopus proteins up regulated (U) or down regulated (D) with FC =/>2 at one or more time points after amputation, and the ratio of U/D, according to biological process category

**Biological process**	**U**	**D**	**U/D**
Signaling	30	13	2.3
Transport	11	19	0.6
Transcription	24	21	1.1
Translation	6	2	3.0
Cytoskeleton	27	20	1.4
Muscle	1	13	0.1
Non-Muscle	26	7	3.7
Extracellular matrix	8	2	4.0
Metabolism	10	12	0.8
Carbohydrate	2	6	0.3
Non-Carbohydrate	8	6	1.3
Cell protection	11	9	1.2
Degradation	7	5	1.4
Cell cycle	12	9	1.3
G1/S	3	4	0.8
M	5	4	1.3
Other	4	1	4.0

Table 4 summarizes the number and percentage of proteins with FC =/>2 as a function of dpa. About 56% of these proteins have FC =/>2 at only one time point after amputation, and this was at 12 dpa in the vast majority of cases. Proteins with FC =/>2 FC at two time points (usually 7 and 12 dpa) or 3–4 time points (usually 5, 7, and 12 dpa) comprised about 22% each. Table 5 summarizes the number of high FC proteins at each time point after amputation and their U/D ratios. The U/D ratios in each biological process category either fluctuated slightly or showed a rise in these ratios from 1-12 dpa. We reasoned that these 275 proteins would be the ones most likely to be involved in fibroblastema formation, including hemostasis and re-epithelialization of the amputation surface. Thus in comparing the formation of the *Xenopus* fibroblastema with blastema formation in the axolotl, we focused on the functions of these high FC proteins, the details of which can be found in the Additional file 4. At the same time, however, we paid attention to some proteins with FC < 2 whose function suggested that they might be relevant to the regenerative process.

**Table 4:** Percentage of up or down regulated Xenopus proteins with FC =/>2 at one time point (TP), 2 time points or 3–4 time points after amputation, according to biological process category

**Biological process**	**1 TP**	**2 TP**	**3-4 TP**
Signaling	53.6	14.3	32.1
Transport	42.4	19.0	38.1
Transcription	48.8	20.9	30.2
Translation	66.6	33.3	00.0
Cytoskeleton	67.2	22.4	10.3
Muscle associated	68.8	18.8	12.5
Non-Muscle Associated	66.7	23.8	9.5
ECM	60.0	30.0	10.0
Metabolism	54.8	21.4	9.5
Carbohydrate	50.0	16.7	33.3
Non-Carbohydrate	0.57	33.3	13.3
Cell protection	38.1	14.3	47.6
Degradation	91.7	00.0	7.3
Ubiquit/Proteasome	85.7	00.0	14.3
Other	100.0	00.0	00.0
Cell cycle	33.3	33.3	33.3
G1/S	43.6	28.6	28.6
M	11.1	55.6	33.3
Other	60.0	00.0	40.0

**Table 5:** Number of highly regulated Xenopus proteins up regulated and down regulated at each time point after amputation and U/D ratios (bold)

	**11 dpa**			**5dpa**			**7 dpa**			**12 dpa**		
	**U**	**D**	**U/D**	**U**	**D**	**U/D**	**U**	**D**	**U/D**	**U**	**D**	**U/D**
Signaling	39	44	**0.9**	58	38	**1.5**	52	34	**1.5**	60	35	**1.7**
Transport	30	30	**1.0**	31	36	**0.9**	36	32	**1.1**	35	35	**1.0**
Transcription	38	47	**1.2**	52	44	**1.2**	54	39	**1.4**	57	43	**1.3**
CA	9	12	**0.8**	14	8	**1.8**	14	8	**1.8**	15	9	**1.7**
TFs	18	14	**1.3**	17	22	**0.8**	18	18	**1.0**	20	18	**1.1**
RP	11	21	**0.5**	21	14	**1.5**	22	13	**1.7**	22	16	**1.4**
Translation	22	43	**0.5**	48	22	**2.2**	57	17	**3.4**	59	15	**3.9**
RPs	16	30	**0.5**	33	16	**2.1**	40	12	**3.3**	42	10	**4.2**
TLFs	6	13	**0.5**	15	6	**2.5**	17	5	**3.4**	17	5	**3.4**
Cytoskeleton	34	43	**0.8**	45	50	**0.9**	48	49	**1.0**	60	43	**1.4**
MPs	6	14	**0.4**	5	23	**0.2**	4	23	**0.2**	4	23	**0.2**
NMPs	28	29	**1.0**	40	27	**1.5**	44	26	**1.7**	56	20	**2.8**
ECM	24	12	**2.0**	30	7	**4.3**	28	8	**3.5**	28	11	**2.5**
Metabolism	39	53	**1.4**	48	76	**1.7**	45	76	**0.6**	59	74	**0.8**
CM	27	37	**0.7**	18	45	**0.4**	20	45	**0.4**	27	43	**0.6**
NCM	12	16	**0.5**	30	31	**1.0**	25	31	**0.8**	32	31	**1.0**
Protection	41	29	**1.4**	41	26	**1.6**	46	26	**1.8**	57	24	**2.4**
INF	23	10	**2.3**	18	13	**1.4**	18	14	**1.3**	25	12	**2.1**
APO	7	4	**1.8**	5	5	**1.0**	5	6	**0.8**	5	6	**0.8**
CHA	11	15	**0.7**	18	8	**2.3**	23	6	**3.8**	27	6	**4.5**
Degradation	28	28	**1.0**	30	26	**1.2**	32	25	**1.3**	39	24	**1.6**
PRO	11	15	**0.7**	9	13	**0.7**	15	13	**1.2**	17	11	**1.5**
NPRO	17	13	**1.3**	21	13	**1.6**	17	12	**1.4**	21	13	**1.6**
Cell cycle	20	25	**0.8**	21	27	**0.8**	27	23	**1.2**	37	17	**2.2**
G1/S	6	8	**0.8**	5	8	**0.6**	7	7	**1.0**	8	6	**1.3**
M	4	8	**0.5**	8	10	**0.8**	8	9	**0.9**	13	5	**2.6**
Other	10	9	**1.1**	8	9	**0.9**	12	7	**1.7**	16	6	**2.7**

CA = chromatin associated; TFs = transcription factors; RP = RNA processing; RPs = ribosomal proteins; TLFs = translation factors; MPs = muscle proteins; NMPs = non-muscle proteins; CM = carbohydrate metabolism; NCM = non-carbohydrate metabolism; INF = inflammation; APO = apoptosis related; CHA = chaperones; PRO = proteasome associated; NPRO = non-proteasome associated.

### Validation of Xenopus proteomic analysis

**Figure 3:**
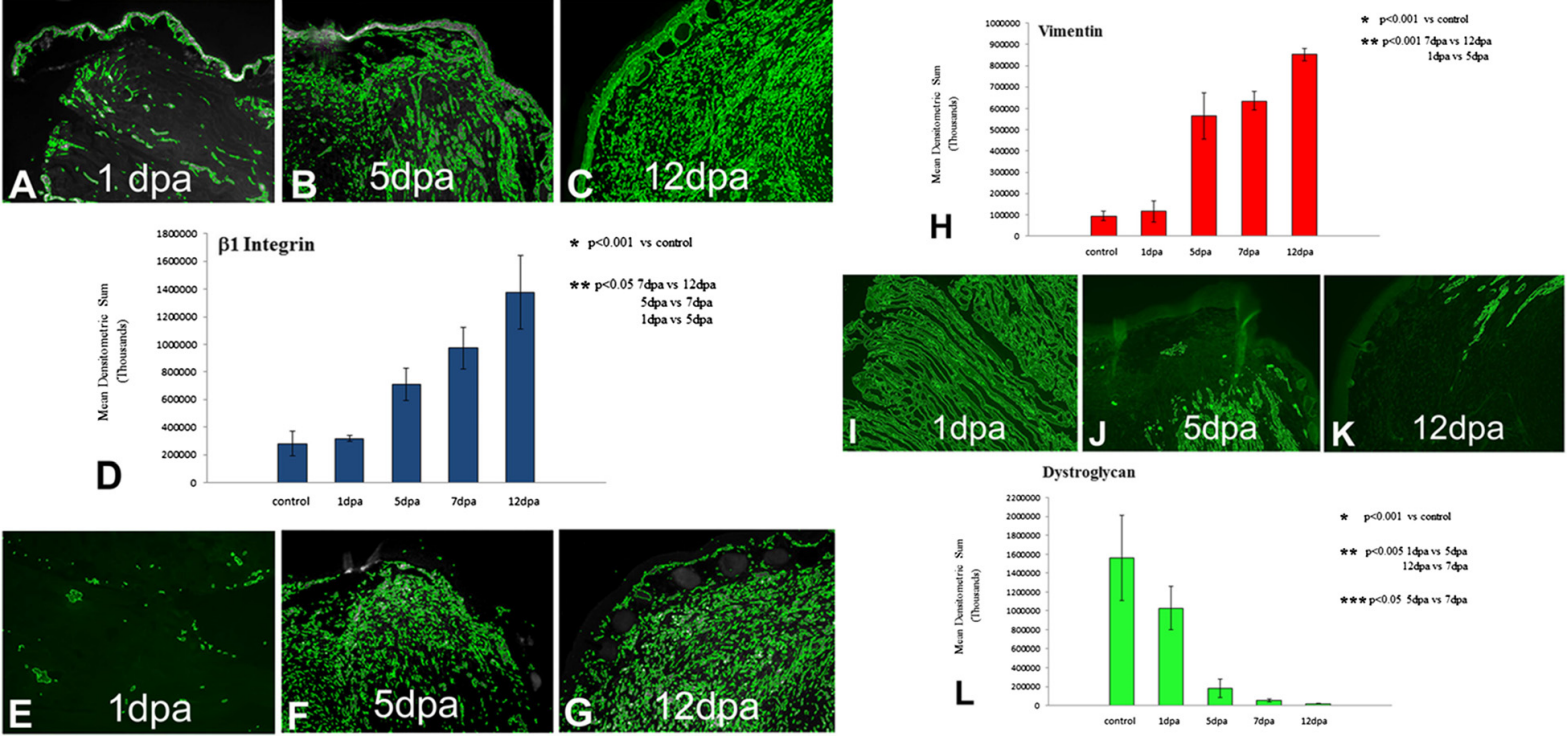
**Validation of LC/MS/MS.** Immunofluorescent antibody staining and mean densitometric sum for β1 integrin, vimentin, and dystroglycan, comparing control, 5 dpa and 12 dpa sections of regenerating froglet hindlimbs. **A-D**, β1 integrin; **E-H**, vimentin; **I-L**, dystroglycan. The 1, 5, 7 and 12 dpa fold changes for each of these proteins were: β1 integrin-1.05, 1.28, 2.18, 2.80; vimentin—1.07, 1.94, 2.30, 3.15; dystroglycan-1.20, -1.40, -1.49, -2.02. The immunofluorescence and densitometry data thus agree well with the LC/MS/MS proteomic data.

We fluorescently immunostained longitudinal cryosections of *Xenopus* limb tissue at each dpa to validate FC data obtained by LC/MS/MS and quantitated the intensity of fluorescence by densitometry (Figure 3). Three proteins were validated: β1 integrin, vimentin, and β2 dystroglycan. The immunofluorescent imaging and densitometry data were in good agreement with the mass spectrometry data.

### Comparison of froglet limb with axolotl limb

We compared the data on fibroblastema formation in *Xenopus* with the data for blastema formation in the axolotl [45] to determine whether there were proteomic differences that could be related to regenerative competence in the axolotl and regenerative deficiency in the froglet. The number of P1 and P2 proteins from the *Xenopus* limb was 2.7 times greater than the axolotl limb. This difference might be related to the greater degree of tissue differentiation and density in the froglet hind limb and the higher complexity in *Xenopus* of systems such as the adaptive immune response that might affect the regenerative process [30]. This difference in complexity is apparent when the expression intensity maps are represented in circular fashion and lines drawn between interacting proteins, as determined by bioinformatic analysis (Additional file 5: Figure S2). Comparison of Table 2 with Table 6 shows that the overall U/D ratio was higher in *Xenopus* (1.3) than axolotl (0.8). The three highest U/D ratios for *Xenopus* proteins up or down regulated on all, or all but one time point were for ECM (6.3), translation (3.1), and cell protection (1.9). The three highest U/D ratios for the axolotl were for translation (4.7), ECM (2.0), and cell cycle (1.8). The lowest ratios (<1.0) in both species were for cytoskeleton (due primarily to depressed muscle proteins) and metabolism (due primarily to depressed carbohydrate metabolism).

**Table 6:** Number of axolotl proteins in each biological process category that are up regulated (U) or down regulated (D) on all dpa or 2 of 3 dpa, and U/D ratios

**Biological process**	**Total**			
**Category**	**Proteins**	**U**	**D**	**U/D**
Signaling	43	23	20	1.3
Transcription	58	26	25	1.0
Chromatin associated	14	8	5	1.6
Transcription factors	22	11	7	1.6
RNA processing	22	7	13	0.5
Translation	20	14	3	4.7
Ribosomal proteins	13	9	2	4.5
Translation factors	7	5	1	5.0
Cytoskeleton	64	14	48	0.3
Muscle proteins	24	0	23	0.0
Non-Muscle proteins	46	14	25	0.6
Extracellular matrix	19	12	6	2.0
Metabolism	33	9	23	0.4
Cell Protection	35	16	16	1.0
Inflammatory-Related	8	6	2	3.0
Apoptosis-Related	13	5	8	0.6
Chaperones	14	5	6	0.8
Degradation	12	4	4	1.0
Cell cycle	13	7	4	1.8
Total		125	149	0.8

The percentage of *Xenopus* proteins with FC =/>2 was nearly 2.4 fold greater (33%) than the axolotl (14%) and the maximum FCs were much higher in *Xenopus*, up to 32 FC, the majority of which were reached at 12 dpa. Of the total of 1034 axolotl plus *Xenopus* proteins up or down regulated at all dpa or 3 of 4 dpa, only 8.9% were common to both species (Table 7). Table 8 lists the shared proteins and those expressed in the axolotl but not *Xenopus*. The highest percentages of shared proteins were found in the categories of translation (18%) and cytoskeleton (17.4%), followed by cell protection (9.5%), metabolism (9.0%), degradation (8.9%), signaling (7.5%), and ECM (7.4%). The least shared percentage of proteins was in the categories of transcription (4.5%) and intracellular transport (2.7%). Cell cycle was the only category that had no shared proteins.

**Table 7:** Number of proteins common to blastema/fibroblastema formation in regenerating axolotl and Xenopus limbs

**Biological process**			
**Category**	**Common**	**Axo**^**+**^**/Xeno**^**-**^	**Xeno**^**+**^**/Axo**^**-**^
Signaling	10 (7.5)	35	87
Transport	2 (2.7)	6	68
Transcription	7 (4.5)	50	98
Translation	15 (18.0)	5	63
Cytoskeleton	25 (17.4)	41	78
Muscle	10 (25.6)	11	18
Non-Muscle	15 (14.3)	30	60
ECM	4 (7.4)	14	36
Metabolism	14 (9.0)	19	121
Cell protection	10 (9.5)	25	70
Degradation	5 (9.0)	6	50
Cell cycle	0 (0.0)	14	56
Total	92 (9.0)	215 (21.0)	727 (70.0)

Numbers in parentheses indicate percent shared.

**Table 8:** **Proteins found in regenerating limbs of *A. mexicanum *** **but not *X. laevis *** **and proteins shared by the two species**

	**Axo**^**+**^**/Xeno**^**-**^	**Axo**^**+**^**/Xeno**^**+**^
***Signaling***	+ APC	YWHAE (-/+)
	+ ARL1	YWHAZ (-/+)
	**+ CCDC88C**	GDI2 (NC/+)
	- CLTCL1	XG28K (+/+)
	- DIXDC1	
	- ECTO	
	+ EPHA7	
	+ EZR	
	+ FHSB	
	+ GNB2L1	
	**+ GPR109B**	
	**+ INV**	
	-IRF6.2	
	**+ IRS4**	
	**+ ISYNA1**	
	**+ ITSN2**	
	-LTBP4	
	**+ NET1**	
	**+ NOS1**	
	**+ OR2AT4**	
	- OR4D10	
	-PPP2CB	
	-PTK6	
	+RAB6B	
	-SYDE2	
	-TBC1D17	
	+TBCK	
	+ TYK-2	
	**+WNT-8A**	
	- DNAH3	**PVALB (-/-)**
	+ DYNC1LI2	DYNLL1 (-/+)
	NC ANXA4	ANXA1 (-/+)
	- ASPH	ANXA2 (-/+)
	**+ ATP11A**	ATP2A3 (-/-)
***Intracellular transport***	+ CACN1A	CASQ1 (+/-)
	+ CACNA2D3	**SLC25A24** (**+/-**)
	- CAMK2D	
	**- HAK**	
	- MYLC2PL	
	- SRL	
***Transcription***		
1. Chromatin associated		
	+ Histone 2A	H2AFY2 (+/+)
		(H2AFX) (+/+)
	NC H2A.ZL1	H1H4B (-/+)
	+ H1H3A	
	- H1H3F	
	+ H2H2AA4	
	-H2BE	
	- H2BF	
	+ HR	
	**-JMJD1B**	
	+ MTA1	
	+ NAP1L1-A	
2. Transcription factors		
	+AHCTF1	FUBP1 (NC/+)
	- ATF1	TRIM 29 (+/+)
	+ CBTF122	
	-E4F1	
	+ Lin 28	
	**- MNT**	
	NEUROD2	
	+ NFATC4	
	**+ NR2C2**	
	+SND1	
	+ SOX6	
	- TAF4	
	+ ZNF259	
	+ ZNF326	
	- ZNF559	
	+ ZNF592	
	+ ZNF644	
	**- ZNF777**	
	+ Kruppel-like factor	
3. RNA processing		
	- CWC15	SFRS1 (-/-)
	+ DDX10	
	- DDX46	
	- FBL	
	- HNRNPF	
	- HNRNPH2	
	- HNRNPH3	
	+ HNRNPL	
	+ HNRNPU	
	+ HNRNPAB	
	+ KHSRP	
	- LSM4	
	+ MATR3	
	- NHP2L1	
	+ RBM	
	- SART1	
	- SFRS12	
	+ SFRS3	
	- SNRPD3	SNRPE (-/+)
	-SRRM1	TARDBP (+/-)
***Translation***		
Ribosomal proteins:	+ RPL31	RPL12 (+/+)
	**+ RPS20**	RPL15 (+/+)
		RPL22 (-/+)
		RPL23 (-/+)
		RPL30 (+/+)
		RPL4 (+/-)
		RPL7L1 (+/+)
		RPLP0 (+/+)
		RPS19 (+/-)
		RPS28 (+/+)
		RPS6 (+/-)
Translation factors:	+ PABPC1	EEF1A2 (+/+)
	- TARSL2	EEF2 (+/+)
	+ ETF1	EIF4A1 (+/NC)
		EIF4B (+/+)
***Cytoskeleton***		
Muscle proteins:	- MYH3	ACTA1 (-/-)
	- MYH4	ACTN3 (-/-)
	- MYH7	DES (-/+ & -)
	- MYH7B	MYBPC3 (-/+)
	- MYL2	MYH13 (-/-)
	- MYL3	MYL1 (-/-)
	- MYL5	MYLPF (-/-)
	- NEB	TNNC2 (-/-)
	- OBSCN	**TNNT3A** (**-/-**)
	**- TM7**	TPM2 (-/-)
	- TPM3	
Non-Muscle proteins:	- ACTBL2	ACTG1 (+/+)
	- ACTN1	DESPLK (NC/+)
	- ACTN4	DESPLK
		Isoform II (-/+)
	+ ACTR2-A	KRT12 (-/+)
	+ EPPK1	KRT19 (+/+)
	+ FLNB	KRT5.5 (-/+)
	- GOLGA1	PLS3 (-/+)
	NC Myo9A	EZR (+/+)
	**+ NAV1**	TUBB2C (+/NC & +)
	**+ SYNE2**	XAKB (-/- & +)
	+ TUBA	XAKC (-/+)
	+ TUBA4B	CytoKer II (-/+)
	+ TUBB4	
	- DNAH3	MVP (-/+)
	- MYH1	DYNLL1 (-/+)
	-MYO1C	
	**+ MYO1E**	
	- Myo5A	
	- PALM2	
	- PDLIM1	
	+ CDH5	
	- CNTNAP4	
	**- FHDC1**	
	+ ST3GAL5	
	+ SCARF2	
	- KPNA2	
	- MYOF	
	+ PCLO	
	**+ PMFBP1**	
	**- SORBS1**	
***ECM***		
	+ Col12A1	**FGB** (**+**/**+**)
	+ ColXIII1	FGG (**+**/**+**)
	+ Col1A1	FN1 (+/+)
	+ Col1A1 pre-prot	MAT2 (+/+)
	- Col2A1	
	- Col4A4	
	+ Col5A2	
	- DCN	
	- EHD4	
	+ FBN1	
	+ MATN4	
	- POSTN	
	**- TINAG**	
	+ TNN	
***Metabolism***		
	- ABCB10	ATP5B (-/NC)
	- CS	COX5A (-/+)
	- ECHS1	GLUD1 (-/+)
	- SLC25A13	
	- ALDOC	**ALDOA** (-/-)
	- ENO3	ALDOB (-/-)
	+ PGAM3	ENO11 (-/-)
		PGM1 (-/-)
		PGK2 (+/NC)
		TPI1 (-/-)
		**PYGM** (-/-)
	+ ACACA	AK1B (-/-)
	+ ALDH6A1	**B3GNT5** (+/-)
	- CA3	BHMT (-/-)
	-CPNE3	
	**+ DAGLB**	
	**+ DHRS4**	
	- DSCR3	
	- NPC2	
	+ PAPPA2	
	- PNPLAS	
	- SGMS1	
	+ SULT1A4	
***Cell protection***		
Inflammation:	- AOX1	PRDX1 (+/+)
	**- CYP2F1**	
	+ GSTP1	
	+ OAS2	
	+PXDN	
	+ TLR6	
Apoptosis:	+ ABTB1	PDCD6IP (-/+)
	- AK2	
	- AKT1S1	
	- BIRC6	
	- FASTKD5	
	+ IL7R	
	+ MAST3	
	+ MICB	
	+ NEK11	
	+ PAIRBP1	
	+ TRAF1	
	- VDAC1	
Chaperones:	- HSP27	CCT2 (+/+)
	+ HSP90AB2P	FKBP10 (-/+)
	+ HSPB3	HSP90AA1 (-/+)
	- PCMT1	HSP90B1 (+/+)
	+ SSR1	PDIA3 (-/+)
	+ TOR1A	PDIA6 (+/+)
	+ HSP90Kda	PPIA (+/+)
	isoform1	P4HB (-/+)
***Degradation***		
	EXOC7	PSMB1 (+/-)
	HACE1	**PRSSL1** (-/+)
	MME	
	PSMD2	
	PSMD7	
	USP3	
***Cell cycle***		
	+ CROCC	
	**+ EVI5**	
	- FUS	
	- LOH11CR2A	
	- MARK4	
	-MMCM3	
	+ NDEL1	
	+ NMW1	
	- PPP1CC	
	- RAN	
	+ TTN	
	+ ULA1	
	- WDR36	
	- XMAP215	

Bold indicates FC =/>2 on one or more dpa; + indicates up regulated; - indicates predominantly down regulated during blastema/fibroblastema formation. NC indicates no change. For the shared proteins, the first symbol(s) in parentheses indicate axolotl, and the second symbol(s) indicate Xenopus.

#### *Signaling*

The U/D ratio of this protein category was slightly higher for *Xenopus* (1.6) than axolotl (1.3) (Tables 2 and 6). The two species shared 7.5% of their signaling proteins (Table 7).

### IP3/DAG pathway and calcium translocation

Inositol triphosphate (IP_3_) and diacylglycerol (DAG) are second messengers generated by G-protein and RTK receptor signaling. In this pathway, inositol-3-phosphate synthase (ISYNA1) catalyzes the synthesis of inositol from glucose-6-phosphate. Inositol is converted to phosphatidylinositol-4, 5 bisphosphate (PIP_2_), which is then cleaved by phospholipase C (PLC) to IP_3_ and DAG. IP_3_ triggers Ca^2+^ release from the endoplasmic and sarcoplasmic reticulum, resulting in the translocation of protein kinase C (PKC) to the plasma membrane, where DAG activates it to regulate transcription [47]. Planarian regeneration has been shown to be dependent on Ca^2+^[48]. In the regenerating axolotl limb, channel proteins in the plasma membrane that mediate extracellular Ca^2+^ influx into the cytosol were up regulated on all dpa (CACNA1A, ATP11A), or at 7 dpa (CACNA2D3), while proteins that translocate Ca^2+^ from the cytosol to the ER/SR (ATP2A3, SRL, ASPH), buffer cytosolic Ca^2+^ during muscle contraction (PVALB), or regulate transport of Ca^2+^ into and out of cells (CAMK2D) were down regulated on all dpa or two of three dpa [45]. In addition to Ca^2+^, Na^+^ influx through sodium channels is obligatory for newt limb regeneration [49] and H^+^ efflux driven by a plasma membrane v-ATPase is obligatory for regeneration of *Xenopus* tadpole tails [50].

Inositol phosphates are among the earliest signals associated with urodele limb regeneration. They are generated from PIP2 within 30 s after amputation of the newt limb and inhibition of their formation by beryllium inhibits blastema formation [51,52]. Consistent with these results, we found that ISYNA1 was up regulated with FC >2 in the proteome of the amputated axolotl limb. By contrast, ISYNA1 was not detected in the froglet limb, and INPP5F, a phosphatase that hydrolyzes PIP_2_, was down regulated, suggestive of deficiencies in the IP_3/_/DAG early signaling pathway. Phospholipase C was up regulated in the froglet limb with FC > 2 at 12 dpa, when the equivalent of an accumulation blastema had been established. ATP2A3, PVALB, and calsequesterin 1 were down regulated (the latter with FC < 2), suggesting that Ca^2+^ efflux does take place from storage compartments in the amputated froglet limb. In general, however, proteins involved in calcium translocation were more predominant in the axolotl than the froglet. The systematic under-expression of up regulated and over-expression of down regulated ion-binding proteins would be useful in dissecting the roles of the various ion-binding proteins involved in blastema formation. It is not known whether H^+^ efflux or Na^+^ influx is essential for limb regeneration in either *Xenopus* or axolotl.

### Annexins

Annexins, calcium-dependent phospholipid binding proteins with important signaling and other functions [53], for review were detected in the regenerating limbs of both axolotl and froglet. The axolotl expressed annexins A1, 2, 4 and 6. With the exception of A2, which was up regulated at 1 and 4 dpa, these showed either down regulation or no change before being up regulated at 7 dpa. None had FC =/>2. The froglet limb expressed annexins A1, 2, 5, 6, 7, 8, and 11, and the ANAXA2 binding partner S100A10A, but not annexin A4. All except A6 were up regulated, with A1, 2, 8, and S100A10 showing FC =/>2 at 12 dpa. Annexin A6 was down regulated with FC <2 at all time points in the froglet limb, whereas A5 was up regulated with FC >2. A6 acts as a scaffold for recruiting PKC to the cytoskeleton, and A5 is a negative regulator of PKC activity, suggesting that although PKC expression is up regulated at 12 dpa in the regenerating froglet limb, it may not be recruited to the plasma membrane and/or its activity is down regulated, again suggesting a potential deficiency in the IP_3_/DAG pathway. By contrast, the axolotl up regulates GNB2L1, a protein that anchors PKC to the plasma membrane, and PKC rises to a peak at accumulation blastema to medium bud [54]. Annexin A5 interacts with type II collagen, an interaction that might play a role in the deposition of ECM in the cartilage spike. Annexin functions of potential importance to blastema formation in both axolotl and froglet are the stimulation of osteoclast formation and bone resorption (A2), and inhibition of enzymes involved in inflammation (A1, A2, A5) [29,55]. Annexins A4 and 6 promote exocytosis in epithelial cells, which may be important for the phagocytosis and elimination of debris by wound epithelium during early blastema formation [56]. Annexins and S100A10 are up regulated in regeneration-competent stage 53 *Xenopus* limb buds as well [44], indicating that their functions are similar in blastema formation of both early tadpole and froglet. Increased expression of several S100 family Ca^2+^-binding proteins has also been observed in the regenerating ear tissue of MRL/Mpj-Fas mice compared to control C57BL/6 J mice [57,58], suggesting that annexins play a role in mammalian regeneration as well.

### NOS1

Another aspect of signaling that differed in axolotl and froglet limbs was the expression of neuronal nitric oxide synthase (NOS1), which catalyzes the synthesis of nitric oxide (NO), a gas that has many signaling functions [59,60]. Grow [33] found that NOS1 transcripts were highly up regulated in amputated stage 53 *Xenopus* limb buds but not in amputated stage 57 limbs, suggesting that failure to produce NO is a factor in the loss of regenerative competence as the *Xenopus* limb bud differentiates. NOS1 was not detected in the froglet limb, confirming this suggestion at the protein level. NOS1 protein was highly up regulated at 1 dpa in the axolotl limb, declining toward control level by 7 dpa, and anti-NOS1 antibody staining showed that it was confined to the wound epidermis. The high expression of NOS1 in axolotl wound epidermis at 1 dpa suggests that NO might activate proteases involved in histolysis [61] and its absence in the *Xenopus* limb might indicate deficiencies in histolysis associated with MMP production [62]. NO might also stimulate axon and capillary regeneration, both of which the accumulation blastema requires for growth.

### Wnt pathway

The Wnt pathway has been implicated in the regeneration of deer antlers [63], zebrafish fin regeneration [64], axolotl limb regeneration [65], and regeneration of limb buds in Xenopus tadpoles [65]. Heat shock-induced expression of the canonical Wnt antagonist Dkk inhibited blastema formation in transgenic *Xenopus* stage 53 limb buds, which are not yet innervated [66], and epidermal-mesenchymal interaction was disturbed by the formation of a thick basement membrane. Wnt3a transcripts were detected in the epidermis of amputated adult *Xenopus* limbs, but expression of Dkk had little effect on epidermal-mesenchymal interactions unless the limbs were denervated, suggesting that Wnt signaling and nerve signaling have redundant roles in fibroblastema formation [67], or that some nerve-dependent function of the epidermis has been compromised.

Wnt8a and APC, components of the canonical Wnt pathway, were detected with FC > 2 and < 2, respectively, during axolotl blastema formation. Inversin, a component of the non-canonical pathway, was up regulated with FC > 2. Inversin targets the Disheveled (DVL) protein for degradation, switching the canonical pathway to the non-canonical pathway [68]. The DVL-binding protein, CCDC88C, a negative regulator of the canonical pathway, was up regulated in the axolotl limb on all dpa, and DIXDC1, a positive regulator of the canonical pathway, was down regulated on all dpa. The presence of both canonical and non-canonical Wnt pathway components suggests that both pathways may be essential for blastema formation in the axolotl limb [65].

No Wnt proteins were detected in our amputated froglet limbs, but BRD7, which activates the canonical Wnt pathway by Disheveled-dependent phosphorylation of GSK3B, was down regulated in the froglet limb with FC > 2. These patterns of protein expression suggest that Wnt signaling, though perhaps not essential for abnormal blastema formation in the froglet limb, may be required for events that result in the formation of a regeneration-competent blastema.

### Receptors and kinases

The expression of receptors and kinases was of interest for signaling. In the axolotl limb, GPR109B, the receptor for nicotinic acid was up regulated with FC > 2, and EPHA7, the receptor tyrosine kinase for the A1 to 5 members of the ephrin A family of ligands was up regulated with FC < 2. EPH and EPH-related receptors have been implicated in neural development [69,70]. With regard to kinases, TYK2, which phosphorylates receptors of the JAK/STAT signaling pathway was up regulated during axolotl blastema formation, whereas PTK6, which phosphorylates STAT proteins, was down regulated on all dpa.

In the froglet limb, the neural receptors NGFR, DLGH4, GPR83.2 and GRIK2 were up regulated, while the neural receptor GABBR2.2 was down regulated, all with FC > 2. Moreover, RUFY3, a protein active in growth cones that has been implicated in axon formation by developing neurons was up regulated with FC > 2 at 7 and 12 dpa. The strong expression of most of these neural receptors conforms to the fact that, as in the axolotl, formation of the froglet fibroblastema is nerve-dependent [21,22,71]. ROR2 and IGF-1R, two receptors involved in chondrocyte differentiation, were up regulated with FC > 2 during fibroblastema formation, perhaps reflecting its propensity for cartilage differentiation. LMBR1 (limb forming region 1), a lipocalin receptor that contains a *shh* DNA-binding domain and is involved in retinoid transport, was up regulated in the fibroblastema. Lastly, Rab family GTPases were up regulated in both axolotl and froglet limbs. This family of proteins plays a critical role in vesicular recycling of receptors, providing another means of regulating signaling.

In *Xenopus*, three kinases were up regulated at 12 dpa with FC > 2, PIK3R4, MAPK1 and STK38. PIK3R4 is a regulatory subunit of the phosphoinositide 3-kinase complex (PI3K) that regulates other proteins through PKB (AKT) and MAPK1 is an extracellular signal kinase (ERK). ERKs phosphorylate transcription factors and are the final step and integration point for Ras pathway intracellular signaling cascades. STK38 (12 dpa) is a negative regulator of MAP3K1/2 signaling. It converts MAP3K2 from its phosphorylated form to its non-phosphorylated form and inhibits autophosphorylation of MAP3K2. Kinases down regulated in the froglet limb were MAPK15 (5, 7, 12 dpa) an ERK that phosphorylates transcription factors, and WDR34 (12dpa), which inhibits the signal transduction functions of MAP3K7 required for TGFβ, BMP, MKK/JNK, Toll-like and IL-1R receptor signaling pathways.

The PI3K pathway phosphorylates and activates the anti-apoptosis protein AKT, whereas MAPK1/2 (ERK1/2) is the substrate for MEK1/2 kinase activity in RTK signal transduction. Suzuki et al. [23] studied these pathways *in vitro* and *in vivo* in amputated froglet limbs transgenic for GFP under control of the *prx1* enhancer, using the inhibitors LY294002, which inhibits the phosphorylation of AKT by PI3K, and U0126, which inhibits the phosphorylation of ERK1/2 by MEK1/2. Activation of the *prx1* gene is an early marker of blastema cells in amputated *Xenopus*[22] and axolotl [24] limbs. In the absence of the inhibitors both pathways are activated within the first 4 dpa, though the ERK pathway is activated slightly later than the PI3K pathway. Inhibition of both pathways repressed GFP expression, decreased the number of BrdU + and PH-H3+ cells, and inhibited cell cycle progression in the limb stumps. Thus, although Ras signaling pathways are activated in amputated *Xenopus* limbs, they appear to be insufficient for normal blastema formation.

### Adaptor proteins

A subset of adapter proteins was detected in the forming blastemas of both axolotl and froglet. The most prominent adapters detected were members of the highly conserved tyrosine 3-monooxygenase/tryptophan 5 monooxygenase activation (14.3.3) family, which contains 7 isoforms. Via binding to phosphoserine/threonine proteins these enzymes integrate many cellular processes such as metabolism, protein trafficking, signal transduction, apoptosis and cell cycle regulation. YWHAZ (zeta) and YWHAE (epsilon) were down regulated with FC < 2 throughout blastema formation in the axolotl limb. By contrast, YWHAZ and YWHAE were up regulated in the Xenopus fibroblastema, YWHAZ with FC > 2 at 12 dpa. Two other members of this family, YWHAG and YWHAQ were also up regulated in Xenopus with FC < 2. Other adapter proteins detected in the fibroblastema but not in the axolotl blastema were WASF4 and PDLIMB, which were up regulated and down regulated, respectively, with FC > 2.

### Chordin

The last aspect of signaling to be considered here is the patterning molecule chordin, which was strongly up regulated at 5, 7, and 12 dpa in regenerating froglet limbs, but was not detected in the regenerating axolotl limb. Chordin is an antagonist of BMP and is known to dorsalize early vertebrate embryonic tissues by binding to ventralizing BMPs and sequestering them in complexes so they cannot interact with their receptors [72]. The up regulation of chordin in the amputated *Xenopus* limb may dorsalize the fibroblastema to create symmetry in the DV axis, thus complementing the AP symmetry caused by the lack of *shh* expression [36].

#### *Transcription*

The U/D ratio for transcription-related proteins in the axolotl blastema was 1.0 (Table 6). The comparable number for the *Xenopus* fibroblastema was 1.4 and 3.1 (Table 2). Shared proteins were 4.5% (Table 7).

### Chromatin associated proteins

The chromatin-associated proteins expressed during *Xenopus* fibroblastema formation were primarily histones or histone binding proteins that regulate the state of chromatin condensation and remodeling (alteration of DNA-nucleosome topology). Four such proteins were up regulated with FC > 2. BTBD17 (12 dpa) is a transcriptional repressor whose *in vivo* function has not been defined. HIST1H1D (12 dpa) is a linker histone essential for chromatin condensation. The high mobility group proteins HMGB2 (12 dpa) and HMGX (12 dpa) are non-histone DNA binding proteins that facilitate cooperative interactions between cis-acting proteins.

Five chromatin-associated proteins were down regulated with FC > 2 in the fibroblastema. ACTL6A (12 dpa) is part of a HAT complex that activates transcription by acetylation of histones H4 and H2A. NCOR1, which was down regulated with FC > 4 at 12 dpa, mediates ligand independent transcriptional repression of thyroid hormone and RA receptors by promoting histone deacetylation and chromatin condensation. SIN3B (1, 5 dpa) represses transcription by serving as a scaffold to tether HDAC enzymes and thus prevent histone deacetylation. LRB (7, 12 dpa) attaches chromatin to the nuclear envelope and helps maintain chromatin structure. POLR1A (1, 5, 7, 12 dpa) is the large subunit of a DNA-dependent RNA polymerase; it had a FC of -5.3 at 1 dpa and -14.36 at 12 dpa. RNA polymerase was not detected in the axolotl.

Several H21 and H2A histones were detected during blastema formation in the axolotl limb, most of which were up regulated. Hairless (HR) in rat functions as a transcriptional co-repressor for thyroid hormone and interacts with histone deacetylases. Two proteins that regulate gene expression by covalent modification of histone proteins, MTA1 and nucleosome assembly NAP1L1-A, were up regulated at all dpa and 4 and 7 dpa, respectively. The histone lysine demethylase JMJD1B (KDM3B) was down regulated on all dpa with the highest FC of all the axolotl proteins, negative 6.8 at 7 dpa. This enzyme specifically demethylates Lys-9 of histone H3. Methylation of this amino acid leads to transcriptional silencing; maintenance or up regulation of KDM3B expression would counter this. The fact that KDM3B is strongly down regulated instead suggests that the genes it regulates are transcriptionally silenced as part of the shift in transcriptional activity leading to repression of genes associated with tissue differentiation and the activation of progenitor cell and more embryonic ECM genes [73-76].

### Transcription factors

In the amputated axolotl limb, most of the 21 transcription factors detected were up regulated at all three or two of three time points. Several of these factors, NR2C2, NFATC4, and SOX6 induce transcription of specific sets of genes. NR2C2 is a nuclear receptor for mineralocorticoids and glucocorticoids, NFATC4 plays a role in inducing cytokine gene expression in T- cells, and Sox6 is required for neurogenic and skeletal regeneration. Of these, only NR2C2 had FC > 2, suggesting that mineralocorticoids and glucocorticoids play an important signaling role in axolotl blastema formation.

Transcription factors with functions related to dedifferentiation are of particular interest. During blastema formation in the amputated axolotl limb transcripts for many of these are up regulated, such as the AEC markers *dlx3* and *sp9*[77,78], the myogenic inhibitor *msx1*[79], a major mediator of stem cell renewal *Notch*), and germ-line specific *PL1* and *PL2* genes, hallmarks for the acquisition of stemness that may function to stabilize the genome by repressing movement of retrotransposons [80]. Denervation and morpholino depletion of PL1 and 2 cause the down regulation of FGF8, suggesting that these transcription factors are involved in establishing a nerve-dependent progenitor state in axolotl limb cells [80]. King et al. [32] noted the activation of genes for several retrotransposons in the blastema and fibroblastema of stage 52/53 limb buds and stage 57 *Xenopus* limbs, respectively. One of these retrotransposons was *LINE1,* which is activated in the amputated axolotl limb as well [81].

A four-factor gene cocktail consisting of the pluripotency factors *Oct4* and *Sox2* plus either *Klf4* and *c-Myc* (OSKM) or *Nanog* and *Lin 28* (OSNL) converts human fibroblasts to pluripotent iPSCs [82,83]. Furthermore, the *Sall4* gene, one of the few pluripotency genes to be expressed in tissue stem cells, augments expression of the “fantastic four” [84]. We found that LIN28 protein was up regulated throughout blastema formation in the axolotl [45]. Transcripts for *c-Myc*, *Klf4*, and *Sox2* are also up regulated during newt limb and lens regeneration [85,86]. The axolotl expresses *Nanog* as a monomer that is sufficient to regulate pluripotency in mammalian cells [87], though its expression has not been reported in the regenerating limb. These data are again consonant with a shift in transcriptional activity during axolotl limb blastema formation toward a progenitor cell pattern. Reprogramming of axolotl limb cells produces undifferentiated progenitor cells that are not pluripotent, but except for fibroblasts are restricted to replacing their original cell type [88]. How factors that restrict the reprogramming of axolotl limb cells by pluripotency factors to progenitor cells instead of iPSCs, and allow a subset of fibroblasts to transdifferentiate into other cell types is unknown.

Transcripts for several pluripotency factors have been investigated in amputated stage 52 vs. stage 57 *Xenopus* limb buds and limbs [89-91]. Stage 52 blastemas did not express *Oct4* or *Lin 28,* but *Sall4* and *Sox2* were expressed. Stage 57 fibroblastemas also expressed *c-myc, Sox2* and *Sall4*, but at lower levels. These results suggest that stage 57 fibroblastema cells are only partly reprogrammed compared to stage 52 blastema cells. The *X. tropicalis* genome lacks the *Nanog* gene [87]. We did not detect any of these transcription factors in our *Xenopus* proteomic data. However, we did detect changes in the expression of SALL1 (up regulated) and LHX9 (down regulated) at FC < 2. SALL1 is a transcriptional repressor that might be involved in inhibiting the expression of differentiation genes. LHX9 is a LIM homeodomain protein that with LHX1 and 2 integrates signaling events linking limb patterning and outgrowth in all three axes [92]. The down regulation of LHX9 might contribute to the faulty patterning of the fibroblastema.

A number of other transcription factors with FC > 2 were detected in the *Xenopus* fibroblastema. PIAS4, MEOX2, LRRFIP1, and TAF3 were up regulated at 12 dpa. TAF3 (TFIID) is one of the general transcription factors required for transcriptional initiation by RNA polymerase II. PIAS4 functions as an ezrin-type small ubiquitin-like modifier (SUMO) ligase. It is a negative regulator of transcription in the Wnt, STAT, p53 and steroid hormone pathways. MEOX2 is a target gene of the TGF-β pathway that regulates the development of skeletal, cardiac and smooth muscle and may play a role in the regulation of limb myogenesis. Since the *Xenopus* regenerate does not possess muscle, MEOX2 might regulate muscle repair in the stump region just proximal to the fibroblastema, or regulate TGF-β-driven fibrosis. LRRFIP1 is a transcriptional repressor for genes encoding TNF, EGFR, and PDGFA, and controls smooth muscle proliferation by repressing PDGFA after injury.

Other transcription factors up regulated were SNIP1 (7, 12 dpa), REST-A (5, 7, 12 dpa), FOXD5 (all dpa), and FOX D5-B (all dpa). SNIP1 is a SMAD nuclear interacting protein that inhibits BMP-induced gene responses [93], which are important in limb development and regeneration [64,94], and thus may play an inhibitory role in the formation of a normal blastema in the froglet limb. REST A represses transcription of neuronal genes in non-neural tissues and has been implicated in axon extension. FOXD5-B plays a role in cartilage development and thus might be essential for the chondrogenesis of spike formation. FOXD5, FOXD5-B, and SNIP1 showed exceptional FCs of 4.73, 4.79, and 6.8, respectively, at 12 dpa.

Several transcription factors were down regulated with FC > 2 in the fibroblastema. NF7 (nuclear factor 7) (1 dpa) is a putative maternal transcription factor selectively retained in the cytoplasm from fertilization to mid-blastula in *Xenopus* and functions in DV patterning. Its down regulation may be another factor contributing to the DV symmetry of the cartilage spike. CNOT10 (12 dpa) is a subunit of the CCR4-NOT transcriptional regulation complex that is also an effector of mRNA decay. E2F8 (12 dpa) directly represses a subset of E2F1-dependent cell cycle progression genes. PAX1 (12 dpa) is a transcriptional activator belonging to the PAX family of proteins that interacts with MEOX2 and plays an important role in vertebrate embryonic pattern formation. SIX4.2 (5, 7, 12 dpa) has been implicated in otic, olfactory, and optic neural cell differentiation. Down regulation of these transcription factors potentially could have inhibitory effects on fibroblastema pattern formation.

### RNA processing proteins

The majority of the RNA processing proteins detected in the amputated limbs of both species was heterogeneous nuclear ribonuclear proteins, small nuclear riboproteins and splicing factors. Most of the axolotl RNA processing proteins had FC < 2 and were predominantly down regulated (U/D, 0.5) until 7 dpa, when the U/D ratio flipped to 1.5 (Table 6). The only axolotl RNA processing proteins with FC > 2 were CWC15, a component of the PRP19-CDC5L complex that forms an integral part of the spliceosome, and the dead box protein DDX46; both were down regulated. Dead box proteins are RNA helicases that contribute to the regulation of differentiation by their ability to modulate RNA secondary RNA structure, and thus availability, in processes such as ribosome and spliceosome assembly, translation initiation and RNA splicing.

In the *Xenopus* fibroblastema, RNA processing proteins were more highly up regulated (U/D ratio, 1.5) than in the axolotl blastema (Tables 2 and 6). Several splicing and dead-box proteins were up regulated by the fibroblastema. Proteins up regulated with FC > 2 were BXDC5, HNRNPH1, LOC494754, and LSM14A at 12 dpa. BXDC5 has been implicated in ribosome biogenesis. HNRNPH1 and LOC494754 are proteins that bind to heterogeneous nuclear RNA and are involved in RNA splicing and metabolism. LSM14A functions in the formation of P-bodies, cytoplasmic structures that provide storage sites for non-translating mRNAs. DDX18 and DDX54 were up regulated at 12 dpa. DDX18 plays an important role in cell proliferation and DDX54 represses the transcriptional activity of nuclear repressors. DDX21 was up regulated at 1 and 12 dpa. This is a nucleolar protein involved in the processing of 20s rRNA to 18 s rRNA. LRPPRC (7, 12 dpa) plays a role in the RNA metabolism of both nucleus and mitochondrion. DDX39 was detected in the developing limb buds of *Xenopus* from stages 48–55 [95], but did not appear in our regenerating froglet limbs.

Four processing proteins were down regulated with FC > 2. U2AF1 is down regulated at 5 and 12 dpa, and CCNL2 (cyclin 2) at 7 and 12 dpa; both are splicing factors. CCNL2 is a novel member of the cyclin family. Overexpression inhibits proliferation and differentiation of mouse EC p19 cells and induces them to undergo apoptosis. RNPS1-A and SCNM1 are down regulated at 5, 7, and 12 dpa. SCNM1 functions as a splicing factor; RNPS1-A is part of a post-splicing multiprotein complex that detects exported mRNAs with truncated open reading frames and initiates nonsense-mediated mRNA decay. The FC at 12 dpa for RNPS1-A, CCNL2 and SCNM1 was very high, -5.76, -6.2, and -10.18, respectively.

#### *Translation*

The U/D ratio for translation-related proteins was 4.7 in the axolotl and 3.1 in *Xenopus* (Tables 2 and 6). Eighteen percent of translation-related proteins were shared between axolotl and Xenopus (Table 7). A large number of ribosomal proteins was detected in both axolotl and *Xenopus*, many shared (Table 7). Most ribosomal protein showed FC < 2 in both species. In the amputated axolotl limb, two proteins, RPL7L1 and RPS20, were up regulated with FC > 2. Factors for initiation (PABPC1), binding of mRNA to the ribosome (E1F4B), and translocation of nascent protein from the A site to the B site of the ribosome (EEF2) were down regulated or unchanged at 1dpa in the regenerating axolotl limb, but were up regulated at 4 and 7dpa. Another initiation factor, E1F4A1, was down regulated at 1dpa, returned to control level at 4dpa, and was up regulated at 7dpa. The elongation factor EEF1A2 was up regulated on all dpa. TARSL2, which is involved in tRNA aminoacylation, was up regulated at 1dpa, and down regulated at 4 and 7dpa. Lastly, a translation termination factor, ETF1, was up regulated at 4 and 7dpa. Three non-shared transfer RNA ligases and one tRNA synthetase were up regulated with FC > 2 in the *Xenopus* fibroblastema.

#### *Cytoskeleton*

Overall, cytoskeletal proteins were down regulated, but less so in the *Xenopus* fibroblastema than the axolotl blastema (U/D ratio, 0.9 vs 0.3) (Tables 2 and 6).

### Non-muscle proteins

Non-muscle proteins were predominantly down regulated in the axolotl limb (U/D ratio, 0.6), but up regulated in the *Xenopus* limb (U/D ratio, 2.1). Fourteen percent of the non-muscle cytoskeletal proteins were shared (Table 7). Non-muscle cytoskeletal proteins included those involved in adhesion, intracellular movement, motility, shape, cytoskeletal organization and cytoskeletal-associated kinase signaling. They were predominantly up regulated in the amputated *Xenopus* limb (U/D ratio, 2.1) and down regulated in the amputated limb of the axolotl (U/D ratio, 0.6).

Seven of the non-muscle cytoskeletal proteins in the axolotl had FC > 2, of which five were up regulated at one or more dpa and two were down regulated. The five up regulated proteins were NAV1 (7 dpa), PMFB1 (1, 7 dpa), SYNE2 (7 dpa), MYO1E (1 dpa), and STE3GAL5 (1 dpa). NAV 1 possesses 3' to 5' helicase activity and exonuclease activity. The exact function of this protein is unknown, but is thought to play a role in neuronal development and regeneration. PMFB1 and SYNE2 are involved in the general organization of the cytoskeleton that maintains cytoskeletal spatial organization and cellular morphology. ST3GAL5 catalyzes the synthesis of ganglioside GM3, which participates in the maintenance of cellular morphology, but also integrin-mediated cell adhesion. MYO1E is a non-muscle class I myosin that may be involved in intracellular movement and membrane trafficking. Down regulated proteins were FHDC1 (all dpa) and SORBS1 (all dpa). FHDC1 is involved in cytoskeletal organization and SORBS1 is involved in forming actin stress fibers and focal adhesions and is also required for glucose-stimulated insulin transport. A number of keratins were expressed with FC < 2. Keratin 12, keratin 5.5 (larval keratin), and keratin XAK-C were down regulated at 1 and 4 dpa before being up regulated at 7 dpa, keratin 19 was down regulated at 1 dpa and up regulated at 4 and 7 dpa, and keratin XAK-B and keratin type II were down regulated at all time points.

In the amputated *Xenopus* limb, 33 proteins were up regulated with FC >2. Thirteen of these were epithelial keratins: KRT13, KRT19, KRT5.2, KRT6, XAK-A, XAK-C, KRT 14, keratin-3, type I cytoskeletal 51 kDa, all up regulated at 12 dpa; KRT15 (7, 12 dpa), keratin type II (7, 12 dpa), and two other intermediate filaments, vimentin (VIM1) (7, 12 dpa) and MGC84118 (12 dpa). KRT14 was the most highly up regulated with FC of 4.5. The remaining highly up regulated *Xenopus* proteins are implicated in actin binding or polymerization and adhesion. PAFAHB1-B was the most highly up regulated of these, 6.48 at 12dpa. This protein enhances dynein-mediated microtubule sliding by targeting dynein to the microtubule plus end. It may play a role in the migration of fibroblasts during wound healing and by extension, in fibroblastema formation. *Xenopus* proteins involved in functional or structural (cell junctions) adhesion were desmoplakin (DSP) (12 dpa), integrin alpha chain V (ITGAV) (12 dpa), NF2 (12 dpa), DSCAM (1, 5 dpa) and integrin beta 1 (ITGB1) (7, 12 dpa). NF2 (neurofibromin) is similar to the ezrin, radixin, moesin (ERM) family that is thought to link cytoskeletal components to cell membrane proteins. Ezrin itself was also up regulated, but by much less than 2.

The *Xenopus* non-muscle proteins down regulated were EML1 (7, 12 dpa) and INTU (12 dpa), both involved in microtubule assembly and organization and the ATP and actin binding proteins FHOD1 (12 dpa), STK35 (5,7, 12 dpa), TMSB4X (12 dpa), GAP43 (5, 7, 12 dpa), TLN (12 dpa) and EPB4 (7 dpa). FHOD1 is required for assembly of F-actin structures such as stress fibers and plays a role in cell elongation by coordinating the organization of actin fibers and microtubules. Talin and EBP4 are adhesion proteins. Talin plays a significant role in assembling actin filaments with integrins to link the cell membrane to ECM constituents. TMSB4X (thymosin β4) binds to actin monomers to prevent their polymerization, and is thus involved in cell proliferation, migration, and differentiation. Thymosin β4 administered to infarcted mouse hearts also improves cardiomyocyte survival by stimulating production of higher levels of the survival kinase PKB (Akt) and is essential for the enhanced regeneration of *Xenopus* limbs implanted with larval progenitor cells [96,97]. Down regulation of thymosin β4 might thus impair limb cell survival and dedifferentiation in *Xenopus*. GAP-43 is a nervous system-specific membrane adaptor protein that enables the association of phosphatidylinositol-4-5 bisphosphate and PIP2 with actin to facilitate actin polymerization. It is expressed at high levels in neuronal growth cones and is considered to be a crucial component of an effective axon regenerative response. Its down regulation suggests that the axon growth response in the amputated *Xenopus* limb may be less than optimal for effective neural-epidermal interaction.

### Muscle proteins

Sarcomeric cytoskeletal proteins were heavily down regulated, with a U/D ratio of zero in the axolotl limb and 0.1 in the *Xenopus* limb (Tables 2 and 6). Nearly 26% of the sarcomeric proteins of regenerating limbs are shared by the two species (Table 7). In the axolotl, down regulation of sarcomeric proteins on all or two of three dpa, many with FC > 2, is consistent with evidence for the cellularization of myofibers into mononucleate cells that undergo dedifferentiation [52,98-100]. Recently, genetic marking studies have indicated that muscle progenitor cells in the axolotl limb blastema are derived solely from satellite cells, not myofiber dedifferentiation, in contrast to the newt limb, where they are derived solely by myofiber dedifferentiation [101]. The negative FC in sarcomeric proteins is also consistent, in both axolotl and *Xenopus*, with the simple degradation of damaged muscle in a hypoxic environment.

*Xenopus* muscle harbors satellite cells, but the fibroblastema does not contain muscle-forming cells, even though it is supportive of satellite cell differentiation into muscle [102]. We do not know if myofibers in the amputated froglet limb undergo fragmentation and dedifferentiation into mononuclear cells, as appears to happen in urodeles. The source of the cells making up the fibroblastema (periosteum, dermal fibroblasts, Schwann cells, or some combination is unclear. The extent of dedifferentiation of the fibroblastema cells is also unclear. Our histological observations, as well as those of others [6,20], indicate that the cells of the fibroblastema have a fibroblastic rather than mesenchymal morphology, and that these cells begin differentiating into chondrocytes as they accumulate under the wound epithelium.

#### *Metabolism*

##### 

**Carbohydrate metabolism** Recently, Shyh-Chang et al. [103] found that reactivation of the *Lin28a* gene in a dox-inducible Lin28a transgenic mouse enhanced epidermal hair regrowth, digit repair and ear tissue repair. This enhancement was correlated with up regulation of IDH3B, SDHA, NDUFB3 and NDUFB8, enzymes involved in glycolysis and OxPhos, and was prevented by OxPhos inhibition, suggesting that LIN28a enhances regeneration and repair by up regulating glycolysis and OxPhos metabolic reactions. Consistent with this idea, Gorsic et al. [104] detected significant up regulation of the transcripts for cytochromes b and c and intense antibody staining to these cytochromes in the epidermis and underlying tissue of 4 dpa regenerating axolotl limbs. By extension, the LIN28 protein might promote blastema formation through a similar up regulation of glycolysis and OxPhos enzymes and thus ATP production.

Our proteomic data, however, are not consistent with this idea. Carbohydrate metabolism was strongly down regulated in both axolotl and *Xenopus* amputated limbs, parallel with the down regulation of muscle cytoskeletal proteins. The U/D ratio was 0.7 in the froglet limb and 0.4 in the axolotl limb. Several enzymes of the citric acid cycle and OxPhos were detected in both the regenerating axolotl and Xenopus limb, including ATP5a enzymes, and COX-5a, but virtually all were down regulated throughout blastema and fibroblastema formation. The OxPhos enzymes up regulated in the Lin28 transgenic mouse [103] were not detected during blastema formation in the regenerating axolotl limb, but were detected in *Xenopus* where they were down regulated throughout fibroblastema formation. Our data indicate that formation of both the axolotl blastema and the froglet fibroblastema requires the depression of glycolysis and OxPhos. This suggests that if the function of LIN28 in axolotl blastema formation is to reprogram cellular bioenergetics, such reprogramming is the opposite of what is observed in mammalian cells. Alternatively, or in addition, LIN28 may be involved with other pluripotency transcription factors to silence differentiation genes and activate genes characteristic of progenitor cells, as outlined under.

##### 

**Transcription factors** The parallel down regulation of muscle proteins and proteins involved in carbohydrate metabolism is not surprising since muscle is the most abundant and metabolically active tissue in the limb, and carbohydrate metabolism would simply be reflecting the damage to the muscle done by amputation. Reduced carbohydrate metabolism is consistent with previous studies on regenerating urodele limbs that showed a marked decrease in O_2_ usage during formation of the avascular blastema [105], for review, and the histochemical absence of citric acid cycle enzymes [106,107], for reviews. The up regulation of NOS1 in the regenerating axolotl limb, particularly at 1dpa, might play a role in metabolic depression, since NO inhibits glycolysis and electron transport in skeletal muscle [108]. NOS1 was not detected in the regenerating *Xenopus* limb. The connection between depressed metabolism and blastema or fibroblastema formation in amputated axolotl vs. Xenopus limbs is an area worthy of future exploration.

### Retinoid metabolism

One of the enzymes more strongly up regulated on all dpa in the axolotl limb was DHRS4, which is involved in the reversible reduction of all-trans and 9-cis-retinal. This up regulation is consistent with the important roles retinoids play, not only in metabolism, but also in the patterning of the blastema [109,110]. In the amputated Xenopus limb the retinol binding protein 3 (RBP3) was up regulated with FC < 2 at 5 and 7 dpa. This protein shuttles 11-cis and all-trans retinoids between the retinol isomerase in the pigment epithelium and the visual pigments in the photoreceptor cells of the retina; its function in the metabolism of fibroblastema formation is not clear. RDHL, an oxidoreductase involved in RA synthesis was up regulated in the froglet limb with FC > 2 at 12 dpa, suggesting that there is at least some RA synthesis. Interestingly, McEwen et al. [111] found that the cellular retinoic acid binding protein (CRABP2) and retinaldehyde dehydrogenase (RALDH), which catalyzes the formation of RA from retinaldehyde, were up regulated in amputated stage 52/53 limb buds, but no new RA survived because the RA antagonist Cyp26b was also up regulated.

### Iron metabolism

The iron-binding and transport molecule transferrin, which is required for many metabolic reactions, was up regulated at all time points in the *Xenopus* limb, though with FC < 2. Transferrin is essential for mitosis in the axolotl blastema [112]. We did not detect it during blastema formation in the axolotl, but axolotl blastema cells do not exhibit significant mitosis until the accumulation blastema has formed and the AEC is fully innervated (8), whereas mitotic index is as high as 10% in the blastema at 3 dpa in stage 52–53 limb buds [113].

#### *ECM*

The *Xenopus* and axolotl limbs shared 7.4% of the ECM proteins expressed, primarily proteins of the plasma clot. Of these, FGA/FGB (fibrinopeptide) and FGG (fibrinogen) were up regulated with FC > 2 at 1 dpa. The limbs of both species up regulated fibronectin and matrilin 2 with FC < 2 and down regulated type II collagen coincident with up regulation of type I collagen. These changes are indicative of a shift toward expression of a less structured matrix. The axolotl limb also down regulated collagen IV, a component of the basement membrane of the wound epidermis, and collagen IV was not detected in the *Xenopus* limb, consonant with observations indicating open communication between the wound epidermis and the underlying mesodermal tissues in both species [1,114].

Two other *Xenopus* proteins up regulated with FC > 2 that may be relevant to regeneration were ALPL (tissue non-specific alkaline phosphatase, 12 dpa) and TGFBI (12 dpa). ALPL may play a role in skeletal mineralization. TGFBI (transforming growth factor beta-induced) is an RGD-containing protein induced by TGF-β that binds to collagens I, II and IV and inhibits cell adhesion. The U/D ratio of ECM proteins was 2.0 for the amputated axolotl limb and 6.0 for the amputated Xenopus limb. Since histolysis plays a large role in blastema formation, this differential may suggest that the limb tissue matrix of the *Xenopus* limb is more difficult to break down than that of the axolotl limb.

### Cell protection and protein degradation

The regenerating axolotl and *Xenopus* limbs share 9.5% of the cell protection proteins they express.

#### *Inflammation and Apoptosis*

The amputated limbs of both axolotl and froglet up and down regulated a variety of antimicrobial proteins that defend against infection (Tables 2, and 6; Additional file 1: Table S1). The proteins most strongly down regulated in both axolotl and Xenopus were members of the CYP450 family involved in electron transport and drug metabolism. The limbs of both species also up and down regulate a balance of antioxidant and pro and anti-inflammatory cytokines to minimize tissue damage due to inflammation. In mammals, tissue macrophages produce many of these bactericidal proteins and inflammatory/anti-inflammatory cytokines, and macrophages are abundant throughout formation of the axolotl limb blastema. Ablation of macrophages by clonodate during axolotl blastema formation, but not during blastema growth and differentiation, inhibited regeneration and induced fibrosis [115]. Thus functioning macrophages appear to be obligatory for blastema formation by virtue of a cytokine contribution that prevents fibrosis, the opposite of macrophage function in mammalian wound repair by fibrosis.

The urodele regeneration blastema forms under avascular and thus hypoxic conditions [99,116] that could lead to apoptosis. Mammalian cells avoid hypoxia-induced apoptosis by up regulating hypoxia induced factor 1A (HIF1a), which activates the PI3K-dependent cell survival gene *Akt*, as well as glycolytic enzymes to maintain ATP production [117-120]. Neither HIF1a nor AKT (aka protein kinase B) was detected in the amputated limbs of either axolotl or *Xenopus*, but TUNEL assays indicated little or no apoptosis in regenerating axolotl limbs from 1 dpa on [121,122]. We detected a number of pro- and anti-apoptotic proteins in both axolotl and *Xenopus* that were up or down regulated, the balance of which appears to dictate how much apoptosis occurs and when. Extensive apoptosis occurs within the first 24 hr post-amputation in regenerating tadpole and knifefish tails [123,124], and, like the presence of macrophages in the axolotl limb, is obligatory for regeneration of the tadpole tail [123]. Investigations of apoptosis within the first 24 hr post-amputation in the axolotl and *Xenopus* limb have not been reported. The data on pro- and anti-apoptotic proteins and macrophages suggests a functional link between obligatory apoptosis and these immune cells.

#### *Unfolded protein response*

Chaperones, such as heat-shock proteins and isomerases mediate an unfolded protein response (UPR) to cell stress caused by the accumulation of unfolded proteins within the ER/SR. Such accumulation is due to loss of Ca^2+^ homeostasis, inadequate disulfide bond formation of nascent proteins, or deficient protein glycosylation [47,125,126], for reviews. The UPR reduces the amount of protein translocated into the lumen, increasing protein degradation by proteasomes and exocytotic mechanisms, and increasing the capacity to accelerate protein folding in the ER by up regulating isomerases and chaperones. Failure to refold mis-folded proteins or remove them from the ER results in apoptosis. In the axolotl limb 10 chaperones were detected that accelerate protein folding in the endoplasmic reticulum. Five of these were the heat shock proteins HSPB3, HSP90B1, HSP90AB2P, HSP27, and HSP9AA1. HSPB90AB2P, HSP90B1, and HSPB3 were up regulated with FC < 2, while the other two heat shock proteins were down regulated. Expression of HSP70 transcripts was also detected in the regenerating axolotl limb [127].

The UPR appeared to be stronger in *Xenopus* than axolotl, because the U/D ratio for chaperones is 3.6 in the former compared to 0.8 in the latter. Transcripts for stress-response proteins, particularly HSP60 and HSP90, were over-expressed in both amputated stage 52 limb buds and regeneration-deficient stage 57 limbs [34]. HSP60 is a chaperone involved in the folding and assembly of polypeptides into protein complexes in mitochondria. Thus formation of both the canonical urodele-style blastema and the *Xenopus* fibroblastema appear to require activation of a UPR. Proteins that remain misfolded are degraded by the ubiquitin-proteasome system, as well as by various proteases. Nine percent of degradative proteins are shared between the two species.

#### *Endocannabinoid*

The amputated axolotl limb up regulated DAGLB on all dpa with FC > 2. DAGLB catalyzes the conversion of DAG to 2-AG. This endocannabinoid is required for axonal growth during development and thus may play a role in nerve regeneration but its up regulation is also consistent with previous studies showing an increase in endorphins after amputation of adult newt limbs, suggesting its involvement in pain control during blastema formation [128-130]. DAGLB was not detected in the *Xenopus* limb.

### Cell cycle

The U/D ratio of cell cycle proteins was 1.8 in the axolotl and 1.2 in the froglet.

#### *Xenopus*

Several *Xenopus* DNA replication and repair proteins were up regulated. RNASEH 2A (12 dpa) is a ribonuclease that participates in DNA replication by mediating removal of lagging strand Okazaki fragment RNA primers. Other up regulated proteins that target DNA replication and repair were SUMO2 (12 dpa), UBE2N (12 dpa), and WDHD1 (12 dpa).

*Xenopus* cell cycle progression proteins up regulated with FC >2 were CDC25-1-A (1, 12 dpa), TBRG1 (12 dpa), and TP53BP2 (5, 7, 12 dpa). CDC25-1-A is required for progression from G1 to S by activating cyclin-dependent kinase CDKC2. It is specifically degraded in response to DNA damage to prevent cells with chromosomal abnormalities from progressing through division. TBRG1 and TP53BP2 (tumor protein p53 binding protein 2) impede cell cycle progression at G1 and G2/M, respectively, in response to DNA damage. A central function of TP53BP2 is to impede cell cycle progression at G2/M and stimulate apoptosis by enhancing the DNA binding and transactivation function of p53 on the promoters of genes involved in these processes. After showing no change in level at 1 dpa, TP53BP2 levels rose to 4.0, 5.2, and 14.0 at 5, 7, and 12 dpa, suggesting a strong activation of p53.

Up regulated *Xenopus* proteins required for mitosis were ANIN (7, 12 dpa; cytokinesis), NUMA1 (5, 7 dpa; formation and maintenance of mitotic spindles), PAFAH1B-1B (5, 7, 12 dpa; establishment of mitotic spindle orientation), the helicase RECQL4 (12 dpa; recombination), SASS6 (centrosome duplication), and STAG2 (5, 7 dpa; cohesion of sister chromatids).

Other elements involved in *Xenopus* DNA replication/repair and mitosis were strongly down regulated. The DNA ligase LIG1 (12 dpa), the homologous recombination protein SMC6 (5, 12 dpa), the G1/S checkpoint protein NBN (all dpa); CHEK1 (5, 7, 12 dpa), which mediates G2/M cell cycle arrest in response to DNA damage; and RAD52 (all dpa) and ZMCM6A (12 dpa), two components of the MCM helicase complex that initiates DNA replication, showed strong down regulation, as did the M-phase proteins NEK6 (required for chromosome segregation), TACC3 (helps stabilize the mitotic spindle) and Rac GTPase activating protein, a part of the centralspindlin complex that signals contractile ring formation during cytokinesis.

#### *Axolotl*

Many fewer cell cycle proteins were detected during blastema formation in the axolotl than in *Xenopus*[45]. Five proteins were up regulated on all dpa with FC < 2. These were NME1, a kinase that facilitates the synthesis of nucleoside triphosphates other than ATP, CROCC and NDEL1, proteins contributing to centrosome cohesion and anchoring microtubules to the centrosome, respectively, TTN (titin), a structural molecule for chromosomes, and ULA1 (beta amyloid precursor protein), which forms a heterodimer with UBE1C that can bind and activate NEDD8, an ubiquitin-like protein required for progression through the G2 checkpoint. Several axolotl cell cycle progression proteins were down regulated at all or two of three dpa with FC < 2. Three of these are involved in regulation of microtubule assembly. MAP/Microtubule Affinity Regulating Kinase (MARK4) regulates the transition between stable and unstable microtubules; XMAP215 is a microtubule polymerase that organizes mitotic spindle poles; and Ras-related nuclear protein (RAN) regulates microtubule polymerization during mitosis. The others are the WD Repeat Domain 35 protein (WDR36), which is involved in cell cycle progression; LOH11CR2A, the tumor suppressor and negative cell cycle regulator; FUS, which promotes ATP-independent annealing of complementary single-strand DNAs; PPP1CC, which is a component of the PTW/PP1 phosphatase complex that plays a role in the control of chromatin structure and cell cycle progression from mitosis into interphase, and MMCM3, which is required for DNA replication.

The tumor suppressor protein p53 is activated in the axolotl limb following DNA damage by irradiation or an alkylating agent [131]. The activity of p53 decreases during blastema formation in the axolotl limb, and this decrease is obligatory for blastema formation [132]. Neither TP53BP2 nor p53 was detected in our proteomic screen of blastema formation in the axolotl limb, consistent with the necessity for decrease in p53 activity. The difference in TP53BP2 and p53 activity in regenerating axolotl limbs should be explored by targeted proteomic methods and transcript analysis.

#### *MCM helicase complex*

The picture that emerges from these data is one in which both the axolotl and *Xenopus* up regulate some proteins involved in DNA replication and repair, and cell cycle progression, and down regulate others. The proteins involved, however, were totally different in the two species. Cell cycle was the only biological process category in which there were no shared proteins. Nevertheless, one similarity between the two species is that the RAD52 and ZMCM6A proteins in *Xenopus* and the axolotl protein MMCM3 are each part of the MCM helicase complex that initiates DNA replication, and all were down regulated at all dpa. The down regulation of MCM helicase complex proteins in both *Xenopus* and axolotl is puzzling, given the importance of this complex in initiating DNA replication and the fact that both axolotl blastema and *Xenopus* fibroblastema cells synthesize DNA [23,133,134]. Both species might rely on alternative replication initiators, but then the question is why should this be so? Moreover, there was another cell cycle protein detected during blastema formation in the axolotl that deepens the mystery of how cell cycle regulation contributes to blastema formation [45].

#### *EVI5*

A cell cycle protein strongly up regulated with FC > 2 on all dpa during blastema formation in the axolotl limb, but not in the *Xenopus* limb, was the ecotropic viral integration factor 5 (EVI5), which acts to prevent premature progression through the G2/M boundary by stabilizing EMI1, a protein that accumulates in the nucleus in late G1 and inhibits cyclin A degradation by the anaphase-promoting complex/cyclosome (APC/C) [135]. EVI5 may also play a role in the Hippo pathway, which plays a role in the cell cycle, and cell differentiation and dedifferentiation [136]. Vesicle trafficking regulates the Hippo pathway by controlling the translocation of phosphorylated Yes-Associated Protein (YAP) from the cytosol to the nucleus where YAP acts as a co-activator of Smad-7 to activate cell cycle genes [137]. EVI5 inhibits the trafficking protein Rab 11 [138,139], which could prevent phosphorylated YAP from localizing to the nucleus and acting as a mitotic signal. The EVI5/EMI1 complex is degraded after phosphorylation by Polo-like kinase 1 (PLK1), allowing the cell to enter M. A truncated version of EVI5 becomes associated with the chromosomal passenger complex (CPC), and is essential for cytokinesis [140]. Another protein that stabilizes EMI1 is the prolyl isomerase PIN1, isolated from *Xenopus*[141], but we did not detect this protein or EMI1in either axolotl or *Xenopus*. EVI5 up regulation has also been demonstrated in the cells of the ring blastema that regenerates ear tissue of the LPR/Mpj mouse lost by punch biopsy [142].

During blastema formation in the axolotl limb dedifferentiated cells and resident stem cells enter the cell cycle, but have a very low mitotic index [143]. High levels of EVI5 might delay the mitosis of blastema cells until the system is certain that all necessary DNA repairs are made prior to mitosis. Alternatively, or in addition to this possibility, the G2 arrest afforded by high levels of EVI5 might be linked to the reprogramming of dedifferentiation and/or to reinnervation of the AEC. A period of G2 arrest would insure that the maximum degree of dedifferentiation is sufficient to form the accumulation blastema. In the axolotl and newt blastema the AEC becomes innervated and the blastema vascularized by this stage, establishing the conditions necessary for growth of the blastema by mitosis. Under the influence of a signal from nerve axons the AEC produces a mitogen, the anterior gradient protein (AGP) that releases blastema cells from G2 arrest, causing a dramatic increase in the mitotic index [6]. AGP would bind to its receptor PROD1 on the blastema cell surface, triggering the activation of Polo-like kinase 1 to degrade EVI5/EMI1 and allow entry into M phase.

BrdU and phospho-histone H3 labeling studies have shown both DNA synthesis and mitotic cells in sections of regenerating *Xenopus* limbs 1–4 days post-amputation at a ratio of 300 (BrdU)/30 (phospho-H3) cells per section [23], but mitotic indices for the various phases of *Xenopus* limb regeneration have not been determined. Moreover, we do not know whether AGP is expressed under the influence of the nerve in amputated froglet limbs, or whether it is a factor in the outgrowth of the fibroblastema. The comparative analysis of the role of the neural/epidermal circuit, AGP and other mitogens, cell cycle factors such as EVI5, PIN1, the chromosomal passenger complex (CPC) and other molecular pathways affecting mitosis [45,142] offer a rich opportunity to examine the relationship of the cell cycle to epigenetic reprogramming in the regenerating *Xenopus* froglet and axolotl limb.

## Conclusions

Some major similarities and differences between blastema and fibroblastema formation emerged from the data that call for more focused analysis.

### Similarities

Rab GTPases were up regulated in both axolotl and *Xenopus*, supporting their importance in signaling pathways. Proteins involved in translation, particularly ribosomal proteins, were up regulated, consistent with the need to translate whatever mRNAs are coding for proteins involved in blastema and fibroblastema formation. Four proteins involved in retinoid synthetic pathways, DHRS4 (axolotl), and the LMBR1 receptor, RBP3 and RDHL (*Xenopus*) were all up regulated, suggesting that retinoid synthesis takes place during both blastema and fibroblastema formation. Most significantly, muscle proteins and enzymes involved in carbohydrate metabolism were strongly down regulated in parallel, consistent with muscle being the most metabolically active tissue in the limb.

### Differences

There were several proteomic differences between the axolotl blastema and Xenopus fibroblastema associated with early signaling events, histolysis and dedifferentiation, innervation, cell cycle, chondrogenesis, and patterning that might help explain the poor blastema formation and hypomorphic regeneration of the *Xenopus* limb compared to the axolotl limb.

a. Early signaling

The very early production of IP_3_ and DAG from IP_2_, and the Ca^2+^-driven translocation of PKC to the plasma membrane in the amputated axolotl limb are obligatory for blastema formation, but ISNAY1, the enzyme which synthesizes inositol from glucose-6-phosphate and is up regulated in the axolotl limb, is not detected in the *Xenopus* limb. Furthermore, annexin A6, which acts as a scaffold to recruit PKC to the plasma membrane, is down regulated in *Xenopus*, while annexin A5, a negative regulator of PKC activity is up regulated. These results suggest a deficiency in *Xenopus* IP^3^/DAG second messengers that would compromise downstream regulatory pathways acting on histolysis and dedifferentiation or the activation of stem cells. Since these signals are among the earliest known in amputated limbs, their disturbance would have pleiotropic effects. Furthermore, NOS1, which catalyzes formation of the versatile signaling molecule NO, which is highly up regulated in the axolotl limb, was absent in the *Xenopus* limb. Lastly, Wnt signaling, which is clearly operative in the axolotl limb, appears to be deficient in the *Xenopus* limb, since PIAS4, a negative regulator of Wnt, is strongly up regulated.

b. Histolysis and dedifferentiation

Both axolotl and *Xenopus* limbs up regulated annexin A2, indicating a remodeling of the limb matrix, and both up regulated ECM proteins such as fibronectin, matrilin 2, and collagen I while down regulating collagen II, suggesting a less structured matrix. There was mild up regulation of MMP 7 and 9 in *Xenopus*. However, the U/D ratio of ECM proteins was 6.0 in *Xenopus*, as opposed to 2.0 in the axolotl, suggesting a more complex and potentially more difficult matrix in the *Xenopus* limb to break down by proteolytic enzymes.

Although MMPs are active in the amputated limbs of both axolotl and *Xenopus*, and chromatin-associated proteins appear to be transcriptional silencers in both, histological studies suggest that the *Xenopus* blastema is formed primarily by the migration of fibroblasts activated in the periosteum of the amputated skeletal elements, rather than by histolytic liberation of cells and their dedifferentiation. This view is consonant with our histological observations and the fact that PAFAHB1-B, a protein that in mammals stimulates fibroblast migration during wound repair was highly up regulated. A low degree of dedifferentiation in *Xenopus* is also suggested by the up regulation of non-muscle cytoskeletal proteins (U/D ratio 2.1) as opposed to down regulation in the axolotl (U/D ratio 0.6).

In mammals, LIN 28 appears to stimulate bioenergetic reprogramming by up regulating enzymes involved in aerobic metabolism. LIN 28 is up regulated in the amputated axolotl limb, but was not detected in the *Xenopus* limb. If LIN 28 promotes bioenergetic reprogramming in the axolotl limb, the reprogramming is the opposite of what occurs in mammals, because all components of carbohydrate metabolism were strongly down regulated. LIN 28 more likely collaborates in the axolotl with the pluripotency factors OCT4, KLF4, c-MYC, Sox2, and Sall4 to reprogram limb cells to lineage-restricted progenitor cells. The reprogrammed cells are likely to be fibroblasts, given the recent evidence against myofiber dedifferentiation in the axolotl. Although some pluripotency factors are expressed in the amputated *Xenopus* limb, they seem unable to stimulate the same level of fibroblast dedifferentiation that takes place in the axolotl limb. Instead the fibroblasts simply migrate to form a fibroblastema that differentiates into cartilage. Satellite cells do not contribute to the blastema. Both *Xenopus* and the newt appear to share a mechanism that inhibits the migration of satellite cells into the fibroblastema (*Xenopus)* and blastema (newt), but this mechanism is part of a regeneration-competent process in the newt and a regeneration-deficient process in *Xenopus*.

#### *Reinnervation of the blastema/fibroblastema and the cell cycle*

EVI5 is strongly up regulated during blastema formation in the axolotl limb, but was not detected in the regenerating *Xenopus* limb, suggesting a normal G2 pause for proliferating cells. Neural receptors were up regulated in *Xenopus*, but GAP-43 (growth associated protein), a major component of motile growth cones that plays a role in the induction of axonal and dendritic filopodia, is strongly down regulated. GAP-43 is a crucial component of an effective neural regenerative response; its down regulation may compromise proper reinnervation of the AEC and its signaling functions. In turn, sustained growth of the fibroblastema may be compromised, leading to premature chondrogenesis, a notion congruent with the strong up regulation of the receptors ROR2 and IGF-1R and the transcription factor Fox D5-B, all of which stimulate chondrocyte differentiation. The relationship of innervation, production of AEC signals such as AGP and FGFs, the cell cycle and chondrogenic differentiation is a prime target for comparative analysis of blastema/fibroblastema formation and growth that can be extended to other comparative models as well.

#### *Patterning signals*

Four proteins involved in dorsoventral axial patterning were detected in the *Xenopus* limb and warrant further experimental analysis, two of which were up regulated and two down regulated. The up regulated proteins were chordin and SNIP1. Chordin is a signaling molecule that dorsalizes the early *Xenopus* embryo by antagonizing BMPs. SNIP1 is a transcription factor that inhibits BMP-induced gene responses. The proteins down regulated were NF7 and LHX9. NF7 (nuclear factor 7) is a maternal transcription factor that determines the DV axial pattern of the Xenopus egg and embryo. LHX9 is a LIM family transcription factor that, along with LHX2 and LMX1b, integrates the signaling events that link limb patterning and outgrowth along all three axes. Collectively, the data suggest that the expression patterns of these proteins collaborate to dorsalize the fibroblastema, to give the spike dorsoventral symmetry as well as anteroposterior symmetry.

## Methods

### Animal surgery and tissue collection

All surgical procedures and animal care were carried out according to the Association for Assessment and Accreditation of Laboratory Animal Care (AALAC) standards followed at Indiana University-Purdue University Indianapolis (IUPUI). *Xenopus laevis* froglets (1–1.5 cm nose-cloacal length) were obtained from Xenopus 1, Inc (Dexter, MI, USA). And allowed to acclimate to laboratory conditions for 1–2 weeks. Froglets were anaesthetized in 0.0024% (wt/vol) benzocaine and hind limbs were amputated unilaterally through the mid-tibia/fibula. The tissue removed distal to the amputation site served as the 0-day control. The regenerative tissue, along with a sliver (approximately 1 mm) of stump tissue, was collected post-amputation at 1 day (epidermal wound closure), 5 and 7 days (histolysis and dedifferentiation) and 12 days (accumulation fibroblastema). This time frame, ending with a distal accumulation of fibroblastema cells, is equivalent to the 1, 4, and 7 day post-amputation time points ending in formation of an accumulation blastema that were analyzed for the axolotl hindlimb [45]. The tissues were rinsed in sterile phosphate buffered saline (PBS) and flash frozen for LC/MS/MS proteomic analysis, which was conducted by Monarch Life Sciences (Indianapolis, IN, USA).

### Histology

Limb tissues were treated as described previously [45]. In brief, they were fixed in Bouin's solution for 48 h before washing in 50% alcohol to remove the picric acid. They were then decalcified for 3 weeks in Calci-Clear (National Diagnostics, Atlanta, GA, USA) at room temperature. The limbs were dehydrated in a graded series of alcohols up to 100% (1 hr each), followed by two changes of Histoclear (Fisher Scientific, Houston) for 1 h each. They were then infiltrated overnight with Pararaplast (Fisher Healthcare, A Fisher Scientific Company, Houston, TX, USA), followed by a second overnight infiltration with fresh paraplast. After embedding in a third change of Paraplast, longitudinal sections were cut at 10 μm, processed for staining with hematoxylin and eosin or Mallory’s trichrome, and photographed at 10 × magnification on a Leica microscope.

### Proteomic analysis

#### *Sample preparation*

Five pools of tissue were harvested from each of the control, 1 dpa, 5 dpa, 7 dpa and 12 dpa limbs. Each pool contained six tissues, one from each hind limb of an individual animal. The samples were processed as described previously [45]. In brief, flash-frozen tissues were homogenized in lysis buffer containing 8 M urea and 10 mM dithiothreitol (DTT). The resulting tissue homogenates were further reduced by triethylphosphine, alkylated by iododethanol and digested by trypsin overnight [144]. The peptide concentration in each pool was determinded by Bicinchoninic Acid (BCA) Protein Assay (Bio-Rad, Hercules, CA, USA).

#### *LC-MS/MS peptide separation*

Tryptic digested peptides were analyzed by liquid chromatography- mass spectrometry (LC-MS) as previously described [45]. Twenty μg of digested peptides from each pooled sample was injected in duplicate onto a zorbax 300SB-C18 reverse column (1 mm × 5 cm) in a random order and were run on a Surveyor high performance liquid chromatography (HPLC) system (Thermo-Fisher Scientific). The equipment configuration was maintained identical while performing the injections and data collection. Peptides were eluted with a gradient from 5% to 45% acetonitrile developed over 120 min at a flow rate of 50 μl/min, and effluent was electrosprayed into the linear ion-trap LTQ mass spectrometer (Thermo-Fisher Scientific). Data were collected in the 'Triple-Play' mode (MS scan, zoom scan, and MS/MS scan). The resulting data were filtered (to increase the signal to noise ratio) and analyzed by a proprietary algorithm developed by Higgs et al. [145].

#### *Protein identification*

Using SEQUEST (Thermo-Fisher Scientific, Waltham, MA, USA) and X! Tandem (an open source algorithm provided by The Global Proteome Machine Organization http://www.thegpm.org) database search algorithms, database searches against non-redundant (NR) National Center for Biotechnology Information (NCBI) Xenopus database (updated in September 2007) were performed for peptide sequence identification. A confidence score was assigned to each peptide by q value (false discovery rate). The score was based on a random forest recursive partition supervised learning algorithm. The percentage ID confidence score was calibrated so that approximately X% of the peptides with percentage ID confidence > X% were correctly identified [146].

Proteins were classified according to their peptide identification quality (priority). This priority system assigns identified proteins into four priority groups, as described previously by Higgs [145]: Group 1 (P1), multiple unique peptide sequences and at least one peptide with >90% (q-value = 0.10) peptide identification confidence; Group 2 (P2), single peptide with >90% peptide identification confidence; Group 3 (P3), multiple unique peptide sequences and at least one peptide with peptide identification confidence between 75%-90%; Group 4 (P4), single peptide with peptide identification confidence between 75%-90%. All peptides with confidence less than 75% were filtered out and discarded. Thus, P1 proteins had the highest likelihood of correct identification and P4 proteins the lowest likelihood of correct identification.

#### *Protein quantification and statistical analysis*

Protein quantification was carried out using a non-gel based and label-free proprietary protein quantification technology described previously [145,146]. All measurements on experimental samples reflect up- or down-regulation, or no change, relative to zero day (freshly amputated control tissue) samples. Every peptide quantified had an intensity measurement for every sample. This measurement is a relative quantity giving the area-under-the-curve (AUC) from the extracted ion chromatogram (XIC) after background noise removal. The AUC was measured at the same retention time window (1-min) for each sample after the sample chromatograms had been aligned. The intensities were then transformed to the log base 2 scale for reasons described previously [45]. After log transformation the data were quantile normalized [147]. This normalization removed trends introduced by sample handling, sample preparation, HPLC, mass spectrometry, and possible total protein differences.

If multiple peptides had the same protein identification, their quantile normalized log base 2 intensities were weight averaged proportionally to their relative peptide ID confidences. The log base 2 protein intensities were then fitted for each protein by a separate ANOVA statistical. Finally, the inverse log base 2 of each sample mean was calculated to determine the fold change (FC) between samples. The maximum observed absolute FC was also given for each priority level. FC was computed as mean regeneration group/mean control group. A FC of 1 means no change.

The number of proteins with significant changes for each priority group was calculated. The threshold for significance was set to control the false discovery rate (FDR) for each two-group comparison at 5%. The FDR was estimated by the q value, as stated previously. Thus protein fold changes with a q value less than or equal to 0.05 were declared to be significant, leaving 5% of the determined changes assumed to be false positives.

We calculated the median percentage of coefficient of variance (%CV) for each priority group. Percentage CV values were derived from the standard deviation divided by the mean on a percentage scale. The percentage CV was calculated for replicate variation (technical variation) and the combined replicate plus sample variation.

In constructing biological process categories, only proteins having peptide confidence levels of 90% and above and with FDR < 0.05 were included. Many proteins were identified either by the same sequences or different sequences in priority 1 or 2 or both. To avoid redundancy, the fold changes of priority 1 were used if a protein was present in both the priorities, and average fold change was calculated if it belonged to same priority. If a protein had conflicting expression patterns (up-regulated in one case, but down-regulated in the other) then it was not considered.

#### *Bioinformatic analysis*

Proteins not recognized by the algorithm were manually curated. NCBI blastp [148] was used to match the sequences of hypothetical/novel/unknown/unnamed/Locus (LOC)/NIH Mammalian Gene Collection (MGC) proteins against the 'vertebrata' category in blast (taxid: 7742) to identify their closest neighbors. Only the proteins having 90% peptide ID confidence and with FDR < 0.05 were chosen. Accession numbers, gene names and names of the proteins were obtained from Uniprot or NCBI using the protein IDs obtained in the raw data. GeneCards [149] and Uniprot [150] were used to determine their biological processes. Human Protein Reference Database (HPRD) was used to determine molecular function and primary cellular localization. Cluster 3.0 [151] and Java Treeview [152] software available from Stanford University were used to generate the global intensity expression maps and protein interaction maps were obtained using BioGrid (http://thebiogrid.org/). The images were drawn using CIRCOS [153].

#### *Limitations of proteomic methods*

Like every technology, LC/MS/MS discovery proteomics has limitations. Firstly, it does not readily detect low abundance proteins at the desired confidence levels. Secondly, a given protein more often than not has more than one function, depending on context, and therefore could fit into more than one biological process category. We assigned categories according to what appeared to be their major function as elaborated by Genecards or literature. The sensitivity of discovery proteomic analysis will improve, and will be complemented by an expanding repertoire of proteins that can be individually targeted for analysis. Thirdly, we arbitrarily concentrated on proteins with FC =/>2. While the assumption that the role of proteins showing higher FC is more significant in blastema and fibroblastema formation makes sense, the fact that 33% of *Xenopus* proteins had FC > 2 as opposed to only 14% of axolotl proteins cautions that proteins with FC < 2 may also play important roles.

#### *Validation of LC/MS/MS data*

Validation of β1 integrin, vimentin and β2 dystroglycan was carried out by immunostaining as described previously [45]. These proteins were selected because there were mammalian antibodies available that recognize the Xenopus versions of these proteins and the antibodies had been demonstrated to work well for immunostaining. They were also chosen for their expression profiles, with β2 dystroglycan showing strong down regulation and vimentin and β1 integrin showing gradual strong up regulation. The samples were fixed overnight in 2% paraformaldehyde in 0.8 × PBS and then rinsed with 1.0 × PBS before decalcifying for 30 min in Calci-Clear Rapid (Fisher Scientific, Houston). After decalcification, the samples were cryoprotected by sequential incubation in 10%, 20% and 30% sucrose in 1 × PBS, then embedded in a 50:50 mixture of 30% sucrose and Neg 50 frozen section medium (Thermo-Fisher Scientific, Waltham, MA, USA). Sections were cut at 10 μm on a Leica CM1900 cryostat (Leica, Wetzlar, Germany) and incubated in 1× PBS to remove excess embedding medium, then blocked for 30 min in a solution of 0.01% Tween-20 and 5% milk in tris(hydroxymethyl)aminomethane (Tris)-buffered saline. Sections were incubated over night with antibodies to β1 integrin (clone 8C8), vimentin (clone 147) and dystroglycan (clone 7A10) from the Developmental Studies Hybridoma Bank. The secondary antibody was Alexafluor 488 conjugated goat anti-mouse IgG from Invitrogen.

Immunostained sections were observed under the 20 × objective lens on a Zeiss Axiovert 200 M microscope (Carl Zeiss Microimaging, Thornwood, NY, USA). Sections were obtained from the hindlimbs of three animals for each time point. Six images were collected for each section, from regions located at the tip of the amputated limb to just proximal to the plane of amputation for 5 and 12 dpa samples and across the putative amputation plane in control sections. Mean pixel intensities were calculated for each image by sampling 20 randomly distributed regions of each image using the measurement package of the Axiovision software. Regions of sections containing bone were omitted from analysis, as some bone tissue displayed autofluorescence. Statistical comparisons were performed using analysis of variance (ANOVA). A P value < 0.05 was considered statistically significant.

## Competing interests

The authors declare that they have no competing interests.

## Authors’ contributions

NR, FS: Carried out collection of tissue samples and preparation for proteomic analysis, curation of data, and interpretation of results. DJ: Carried out data curation, bioinformatic analysis and generation of figures. MW: Oversaw the LC/MS/MS procedures at Monarch Life Sciences. NMP: Carried out curation of data and research on individual protein function. TLB: Carried out research on individual protein functions and contributed to the generation of figures. MJP: Provided bioinformatics techniques and resources. DJM: Carried out the immunohistochemical validation, contributed to the generation of figures, and researched individual protein function. XC, JAC, BL: Researched individual protein function. DLS: Conceived the project, analyzed the data, and wrote the manuscript. All authors: Provided critique of the manuscript. All authors read and approved the final manuscript.

## Supplementary Material

Additional file 1: Table S1 List of P1 and P2 proteins in each of the ten biological process categories and subcategories, with fold change at each time point post-amputation. Click here for file

Additional file 2: Figure 1 Global expression intensity maps for the 10 biological process categories at 1, 5, 7 and 12 dpa. **A:** Signaling, Cytoskeleton, **B:** .Intracellular Transport, Transcription, Translation; **C:** Metabolism, Cell Cycle, ECM; **D:** Cell Protection, Degradation Red = up regulation; green = down regulation. Level of fold change (FC) is indicated by color intensity. Accession numbers to the right of columns. Intensities for some proteins can be 7–10 times the highest and lowest intensities shown. Click here for file

Additional file 3 Xenopus proteins up or down regulated with FC =/>2 on one or more dpa. Click here for file

Additional file 4 Narrative description of functions for Xenopus proteins up or down regulated by a factor of 2 or more. Click here for file

Additional file 5: Figure S2 Circos representation of differences in protein expression during blastema formationin the axolotl **(A)** and fibroblastema formation in the Xenopus froglet **(B)**. The outermost circle shows shows protein expression according to biological process. Metabolism is the most over-represented biological process category in the Xenopus data, whereas Cytoskeleton is the most over-represented in the axolotl data. There were no proteins identified in the Transport category in the axolotl compared to 70 such proteins in the Xenopus data. The next circle represents proteins expressed with FC =/>2 (blue) or =/ 4 (pink). The Xenopus data contained a far higher number of proteins with these fold differences, especially in the transcription, cytoskeleton and signaling categories compared to the axolotl data. Progressing inward, the next four circles in Xenopus reflect the fold change difference (red = down regulation; green = up regulation; blue = no change) of proteins at 1 dpa, 5 dpa, 7 dpa, and 12 dpa, respectively. In the axolotl, three circles represent FC in protein expression at 1 dpa, 4 dpa, and 7dpa. The innermost circle represents the connections between the interacting proteins within the Xenopus and axolotl data. A comparison of these interactions indicates that the proteomic composition and protein-protein interactions are much more complex during formation of the fibroblastema in Xenopus. Click here for file
