# Supplementary figures and images for: Proteomic analysis of fibroblastema formation in regenerating hind limbs of Xenopus laevis froglets and comparison to axolotl
Source: BMC Dev Biol. 2014 Jul 25;14:32. doi: 10.1186/1471-213X-14-32 (PMC4222900; doi:10.1186/1471-213X-14-32)

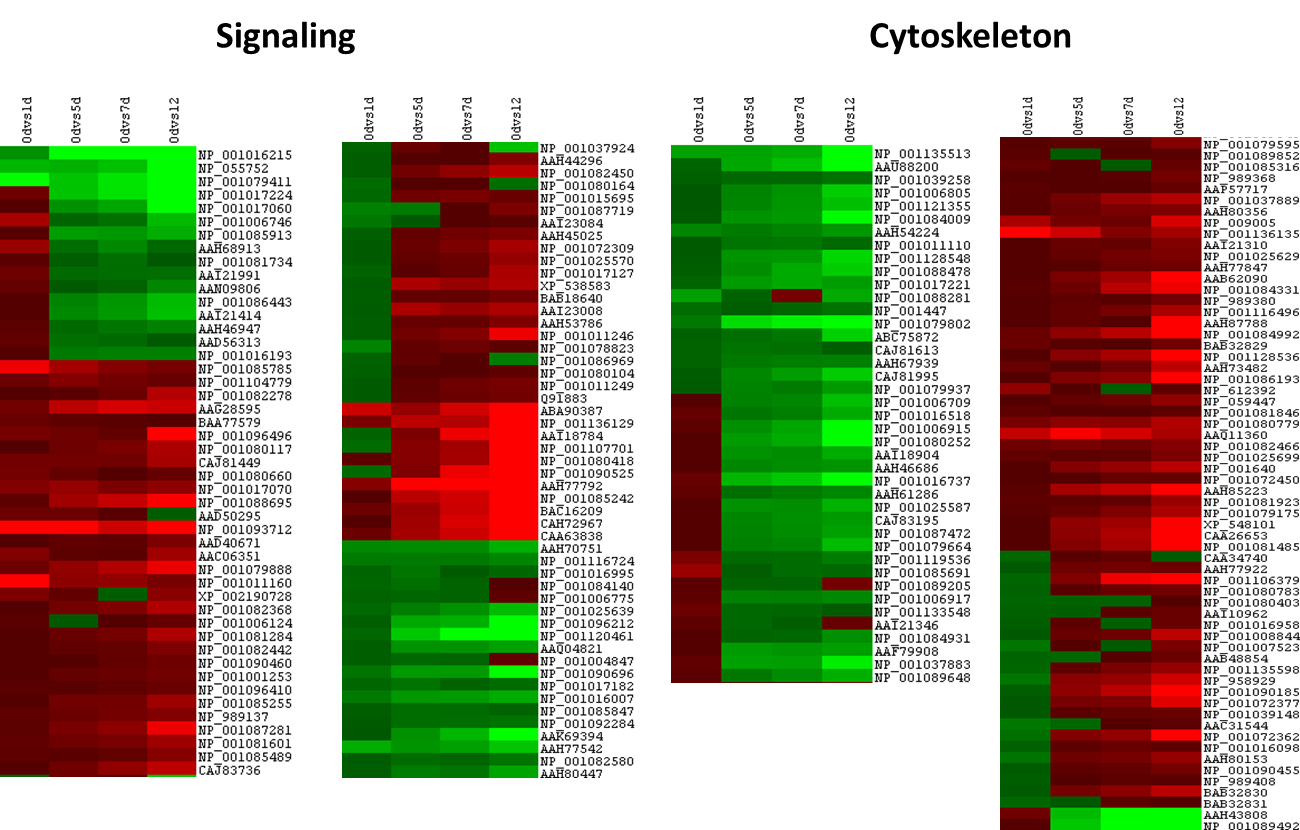

Supplement: Additional file 2: Figure 1 — Global expression intensity maps for the 10 biological process categories at 1, 5, 7 and 12 dpa. A: Signaling, Cytoskeleton, B: .Intracellular Transport, Transcription, Translation; C: Metabolism, Cell Cycle, ECM; D: Cell Protection, Degradation Red = up regulation; green = down regulation. Level of fold change (FC) is indicated by color intensity. Accession numbers to the right of columns. Intensities for some proteins can be 7–10 times the highest and lowest intensities shown. [file 1471-213X-14-32-S2.zip › 2019454066127583_add3.tiff]

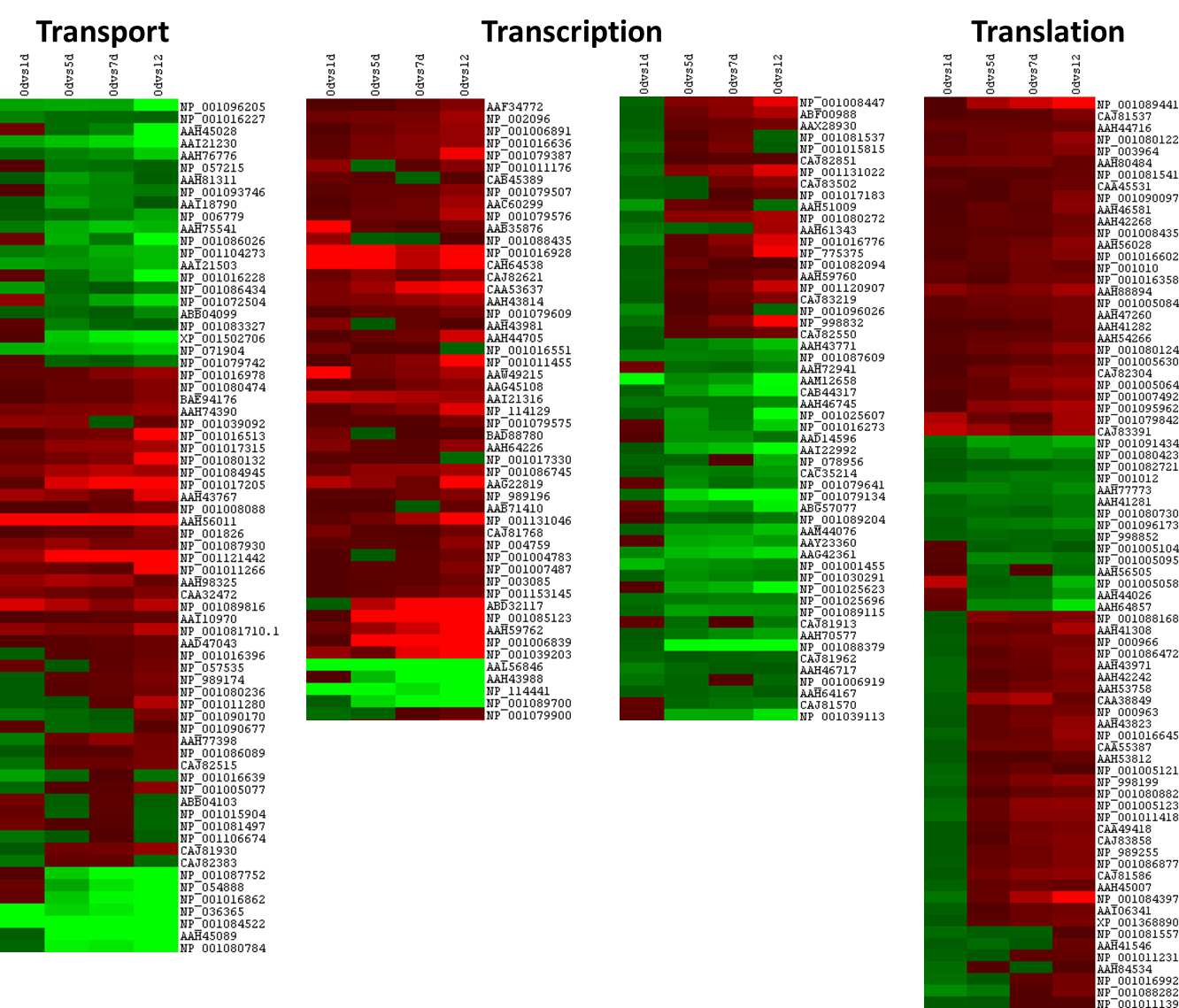

Supplement: Additional file 2: Figure 1 — Global expression intensity maps for the 10 biological process categories at 1, 5, 7 and 12 dpa. A: Signaling, Cytoskeleton, B: .Intracellular Transport, Transcription, Translation; C: Metabolism, Cell Cycle, ECM; D: Cell Protection, Degradation Red = up regulation; green = down regulation. Level of fold change (FC) is indicated by color intensity. Accession numbers to the right of columns. Intensities for some proteins can be 7–10 times the highest and lowest intensities shown. [file 1471-213X-14-32-S2.zip › 2019454066127583_add4.tiff]

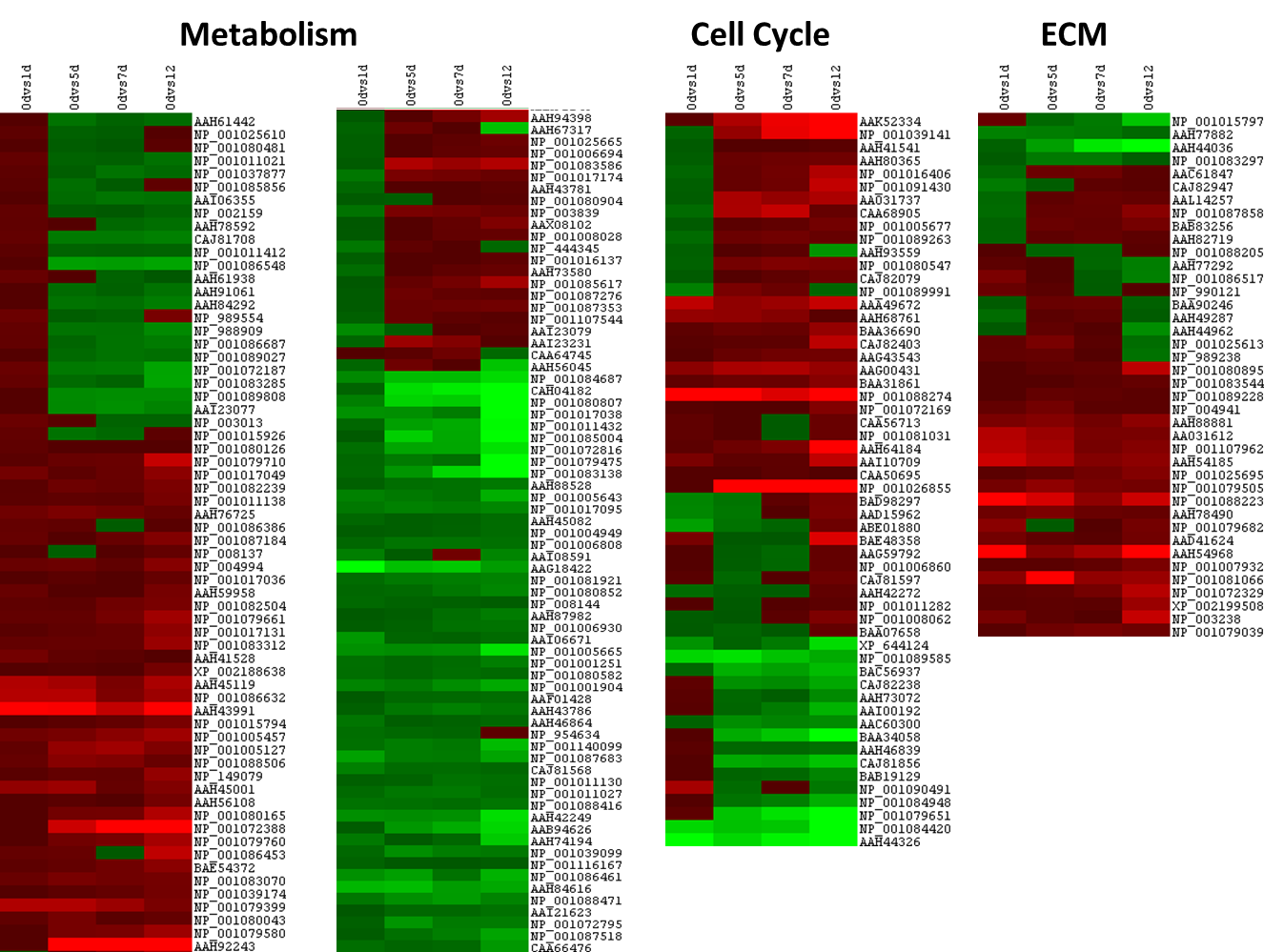

Supplement: Additional file 2: Figure 1 — Global expression intensity maps for the 10 biological process categories at 1, 5, 7 and 12 dpa. A: Signaling, Cytoskeleton, B: .Intracellular Transport, Transcription, Translation; C: Metabolism, Cell Cycle, ECM; D: Cell Protection, Degradation Red = up regulation; green = down regulation. Level of fold change (FC) is indicated by color intensity. Accession numbers to the right of columns. Intensities for some proteins can be 7–10 times the highest and lowest intensities shown. [file 1471-213X-14-32-S2.zip › 2019454066127583_add5.tiff]

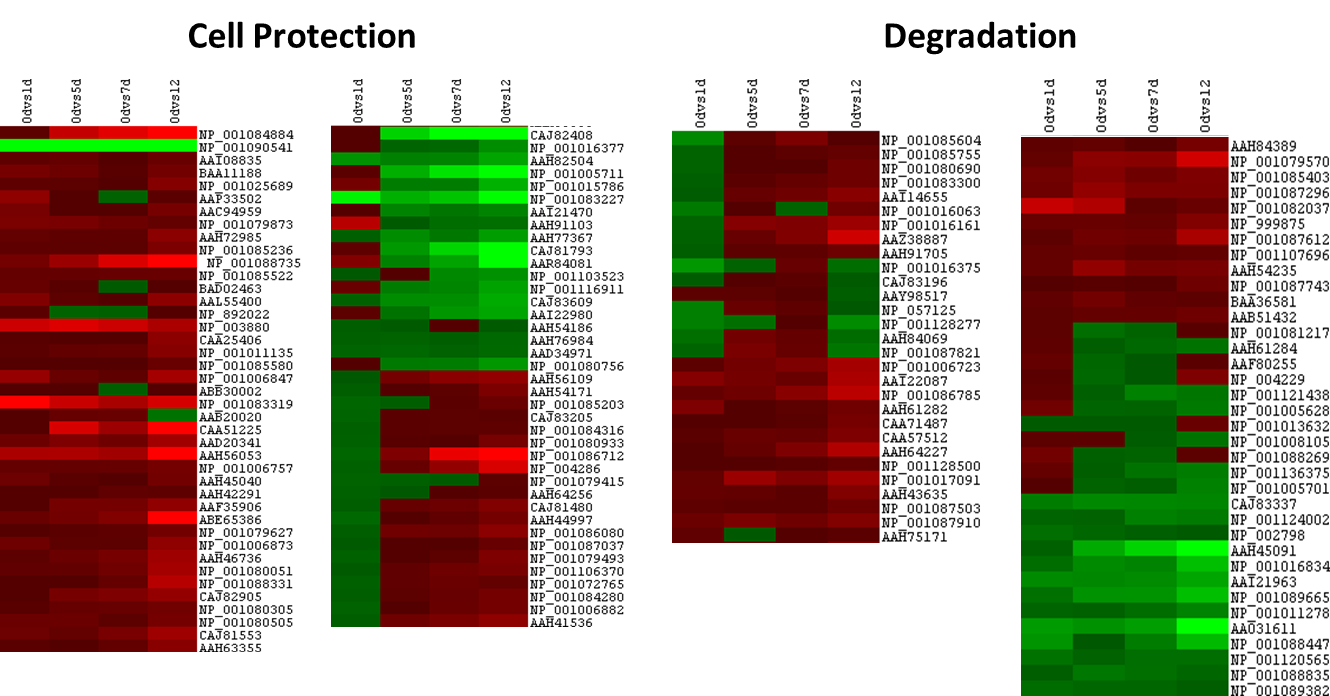

Supplement: Additional file 2: Figure 1 — Global expression intensity maps for the 10 biological process categories at 1, 5, 7 and 12 dpa. A: Signaling, Cytoskeleton, B: .Intracellular Transport, Transcription, Translation; C: Metabolism, Cell Cycle, ECM; D: Cell Protection, Degradation Red = up regulation; green = down regulation. Level of fold change (FC) is indicated by color intensity. Accession numbers to the right of columns. Intensities for some proteins can be 7–10 times the highest and lowest intensities shown. [file 1471-213X-14-32-S2.zip › 2019454066127583_add6.tiff]

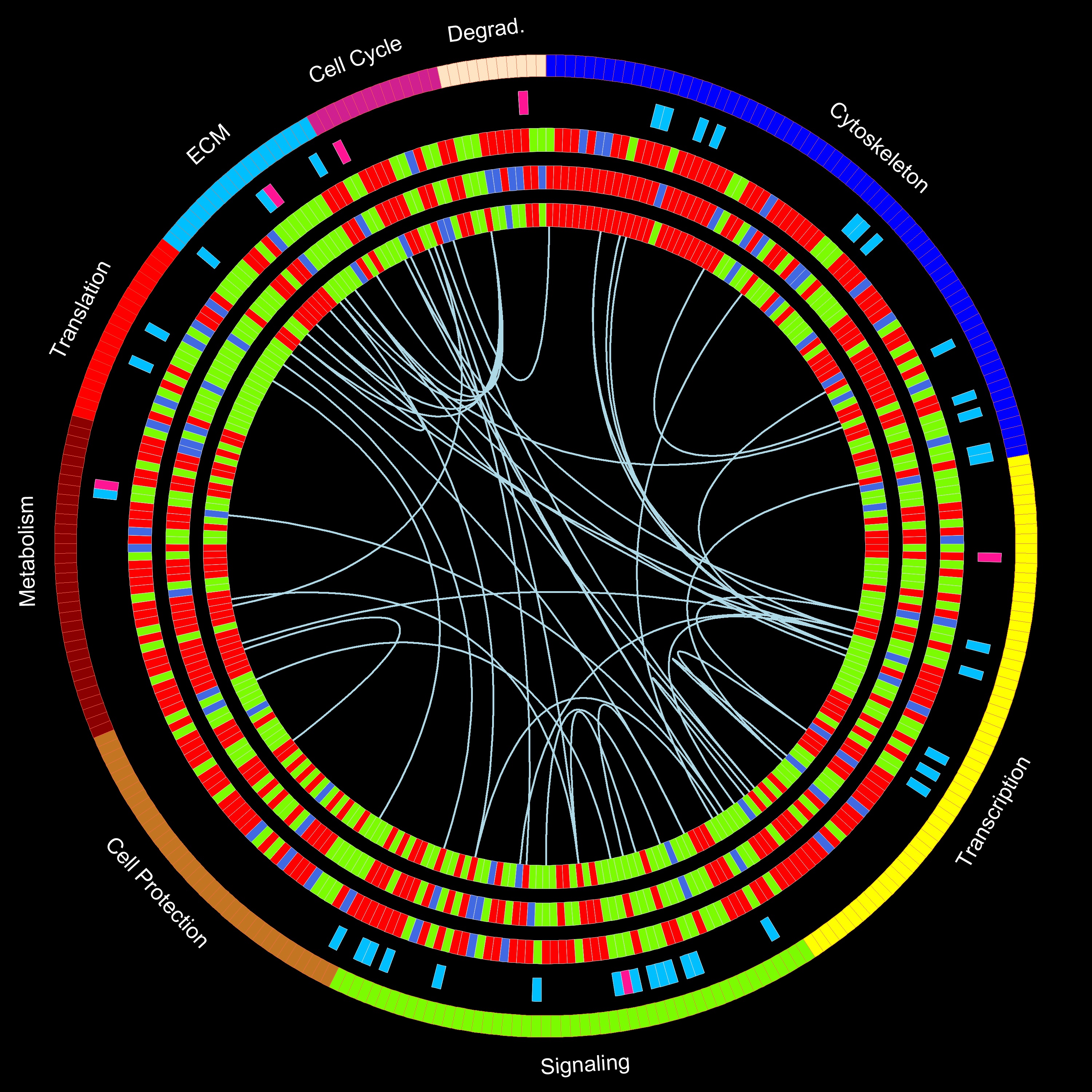

Supplement: Additional file 5: Figure S2 — Circos representation of differences in protein expression during blastema formationin the axolotl (A) and fibroblastema formation in the Xenopus froglet (B). The outermost circle shows shows protein expression according to biological process. Metabolism is the most over-represented biological process category in the Xenopus data, whereas Cytoskeleton is the most over-represented in the axolotl data. There were no proteins identified in the Transport category in the axolotl compared to 70 such proteins in the Xenopus data. The next circle represents proteins expressed with FC =/>2 (blue) or =/ 4 (pink). The Xenopus data contained a far higher number of proteins with these fold differences, especially in the transcription, cytoskeleton and signaling categories compared to the axolotl data. Progressing inward, the next four circles in Xenopus reflect the fold change difference (red = down regulation; green = up regulation; blue = no change) of proteins at 1 dpa, 5 dpa, 7 dpa, and 12 dpa, respectively. In the axolotl, three circles represent FC in protein expression at 1 dpa, 4 dpa, and 7dpa. The innermost circle represents the connections between the interacting proteins within the Xenopus and axolotl data. A comparison of these interactions indicates that the proteomic composition and protein-protein interactions are much more complex during formation of the fibroblastema in Xenopus. [file 1471-213X-14-32-S5.zip › 2019454066127583_add10.jpeg]

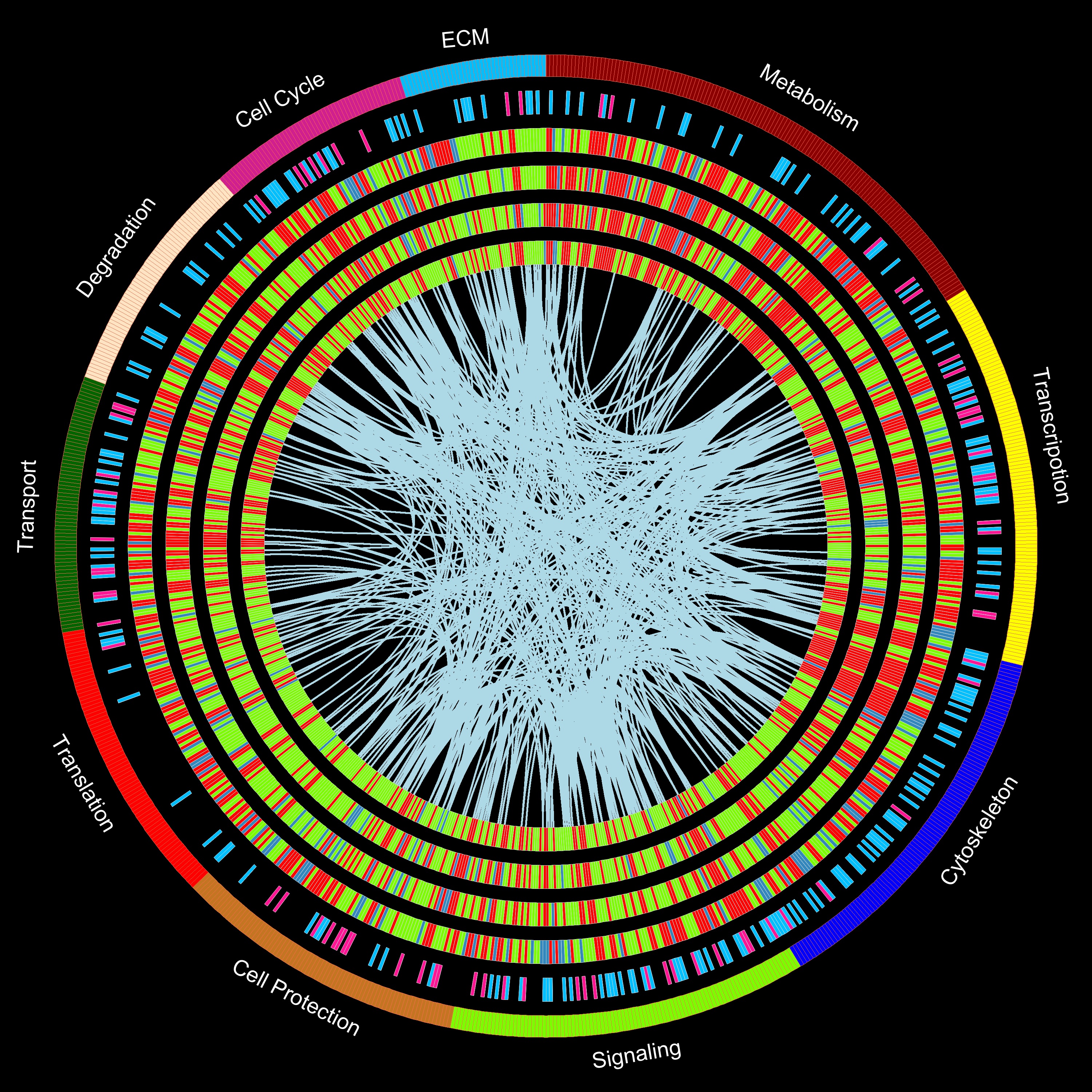

Supplement: Additional file 5: Figure S2 — Circos representation of differences in protein expression during blastema formationin the axolotl (A) and fibroblastema formation in the Xenopus froglet (B). The outermost circle shows shows protein expression according to biological process. Metabolism is the most over-represented biological process category in the Xenopus data, whereas Cytoskeleton is the most over-represented in the axolotl data. There were no proteins identified in the Transport category in the axolotl compared to 70 such proteins in the Xenopus data. The next circle represents proteins expressed with FC =/>2 (blue) or =/ 4 (pink). The Xenopus data contained a far higher number of proteins with these fold differences, especially in the transcription, cytoskeleton and signaling categories compared to the axolotl data. Progressing inward, the next four circles in Xenopus reflect the fold change difference (red = down regulation; green = up regulation; blue = no change) of proteins at 1 dpa, 5 dpa, 7 dpa, and 12 dpa, respectively. In the axolotl, three circles represent FC in protein expression at 1 dpa, 4 dpa, and 7dpa. The innermost circle represents the connections between the interacting proteins within the Xenopus and axolotl data. A comparison of these interactions indicates that the proteomic composition and protein-protein interactions are much more complex during formation of the fibroblastema in Xenopus. [file 1471-213X-14-32-S5.zip › 2019454066127583_add11.jpeg]
