# Supplementary material for: Proteomic analysis of fibroblastema formation in regenerating hind limbs of Xenopus laevis froglets and comparison to axolotl
Source: BMC Dev Biol. 2014 Jul 25;14:32. doi: 10.1186/1471-213X-14-32 (PMC4222900; doi:10.1186/1471-213X-14-32)
Supplement: Additional file 3 — Xenopus proteins up or down regulated with FC =/>2 on one or more dpa. [file 1471-213X-14-32-S3.docx]

**Supplementary file 3: Narrative description of functions for Xenopus proteins up or down regulated by a factor of 2 or more.**

This narrative describes the expression patterns and functions of proteins up or down regulated with FC > 2 during blastema formation in amputated *Xenopus laevis* limbs. In a few cases proteins of interest with FC < 2 are also described and these are identified as such.

**1. Signaling:** The proteins in this category consisted primarily of conserved elements of G protein-coupled receptor systems that have both short and long-term effects on the cell, including G-proteins, small GTPases, kinases and phosphatases, adaptor proteins, calcium binding proteins and enzymes such as phospholipase C that catalyze formation of second messengers. In addition, there were elements of the Ras/MAP kinase pathway used by receptor tyrosine kinases (RTKs), and elements of the Wnt and Notch pathways.

***Receptors (7):*** LMBR1 1 dpa, FC 5.7), GPR83.2 (7, 12 dpa), CSF3R (5, 7, 12 dpa), NGFR (5, 7, 12 dpa), DLGH4 (5, 12 dpa), GRIK2 (7, 12 dpa), and ROR2 (7, 12 dpa) were up regulated. LMBR (Limb Region 1**)** is a putative plasma membrane lipocalin receptor. Lipocalins are a family of proteins that transport small hydrophobic molecules such as steroids bilins, retinoids and lipids. LMBR1 contains a highly conserved cis-acting regulatory module for *shh* within one of its introns. Presumably, disruption of this genic region can alter sonic hedgehog expression and affect limb patterning, but it is not known if this gene functions directly in limb development.

GPR83.2 is a G protein-coupled receptor, perhaps for neuropeptide Y. The nerve growth factor receptor, NGFR is a low affinity receptor that can bind NGF, BDNF, NT-3, NT-4 and mediate survival or death of neural cells. GRIK2 is an ionotropic glutamate receptor. DLGH4 is a molecular scaffolding protein that binds and clusters N-methyl-D-aspartate receptors at neuronal synapses; it may be involved in guanine nucleotide-mediated signal transduction pathways.

ROR2 is a RTK that may be involved in the early formation of chondrocytes. Both NGFR and ROR2 have exceptionally high FC at 12 dpa (6.47 AND 6.94, respectively). It is possible that NGFR up regulation mediates the effects of neurotrophic factors in blastema formation and growth, and that ROR2 plays a role in formation of the cartilage collar and differentiation of the cartilage spike. CSF3R is a receptor for granulocyte colony stimulating factor. Another receptors up regulated < 2, but which might play a role in fibroblastema formation is insulin like growth factor 1 receptor (IGF1R), a RTK that mediates the actions of insulin-like growth factor 1 (IGF1).

The only receptor down regulated >2 was GABBR2.2 (12 dpa), a G protein coupled receptor for GABA that inhibits neuronal activity. Another down regulated receptor associated protein, MPP6 (12 dpa), is a membrane associated guanylate kinase that clusters receptors by forming multiprotein complexes containing distinct sets of transmembrane, cytoskeletal and cytoplasmic signaling proteins.

***Adaptor proteins (3):*** Two up regulated adaptor proteins were WASF4 (12 dpa) and YWHAZ (12 dpa). WASF4 is involved in signaling from RTKs and small GTPases to the actin cytoskeleton. YWHAZ was the only one of four tyrosine 3-monooxygenase/tryptophan 5-monooxygenases to be up regulated with FC > 2; these proteins mediate signal transduction by binding to phosphoserine-containing proteins in multiple signaling pathways. They represent an integration point for proliferative, survival, apoptotic and stress signaling pathways. Another adapter protein up regulated with FC <2 was fibroblast growth factor receptor substrate 2 (FRS2). FRS2 links activated FGF and NGF receptors to downstream signaling pathways,.

The one down regulated adapter protein was PDLIM (12 dpa), which assembles actin filament complexes and binds protein kinases.

***G-Proteins and Small GTPases (7):*** Many receptors are associated with G-proteins that modulate or transduce signals by exchanging GDP for GTP on their alpha subunit. Exchange of GDP for GTP activates the receptor, while the reverse exchange deactivates it. Other proteins involved in GDP/GTP exchange are the small GTPases that hydrolyze GTP to GDP, accelerated by GTPase activating proteins. The Ras superfamily is a well-known group of G proteins. Within the superfamily, Ras proteins regulate proliferation, Rho proteins regulate cell morphology, Ran proteins regulate nuclear transport, and Arf proteins regulate vesicle transport.

One G-protein and three small GTPases were up regulated. The G-protein was GNAI2i (7, 12 dpa) and the small GTPases were RASGEF1c (12 dpa), Rho-GDI like protein (7, 12 dpa) and RAC 3 (1, 5, 7, 12 dpa). GNAI2i inhibits adenylate cyclase in response to beta-adrenergic stimuli, and showed a FC of 4.8 at 12 dpa. RASGEF1c is a guanine exchange factor, Rho-GDI is a GDP dissociation inhibitor, and RAC3 is a small GTPase that binds to effector proteins regulating cellular responses such as cell spreading and formation of actin-based protrusion. RAC 3 up regulation stood out in particular, with a FC > 3 on three of four dpa. Another up-regulated protein was LYPLA1 (12 dpa), a lysophospholipase that hydrolyzes fatty acids from S-acylated cysteine residues in G proteins, thus acting on biological membranes to regulate multifunctional lysophospholipids.

Two small GTPases were down regulated. CHN2 (12 dpa) is a Rho GTPase activating protein whose activity is regulated by binding to phospholipid and diacylglycerol (DAG), and SBF2 (12 dpa) is a guanine exchange factor.

***Kinases and Phosphatases (7):*** Three kinases were up regulated. PIK3R4, a regulatory subunit of the phosphoinositide 3 kinase (PI3K) complex that regulates other proteins through PKB (AKT), and MAPK1, an extracellular signal kinase (ERK), were both up regulated at 12 dpa, PIK3R4 strongly so. ERKs phosphorylate transcription factors and are the final step and integration point for Ras pathway intracellular signaling cascades. STK38 (12 dpa) is a negative regulator of MAP3K1/2 signaling. It converts MAP3K2 from its phosphorylated form to its non-phosphorylated form and inhibits autophosphorylation of MAP3K2

Down regulated kinases were MAPK15 (5, 7, 12 dpa) an ERK that phosphorylates transcription factors, and WDR34 (12dpa), which inhibits the signal transduction functions of MAP3K7 required for TGFβ, BMP, MKK/JNK, Toll-like and IL-1R receptor signaling pathways.

Two phosphatases, IMPAD1 (inositol monophosphatase 3) (5, 7, 12 dpa) and INPP5F (inositol polyphosphate 5-phosphatase F) (1, 5, 7, 12 dpa) were down regulated, particularly at 12 dpa. IMPAD1 is an enzyme of the inositol phosphate second messenger-signaling pathway that catalyzes the hydrolysis of phosphoadenosine phosphate to adenosine monophosphate. It may play a role in the formation of skeletal elements derived through endochondral ossification, possibly by clearing adenosine 3', 5'-bisphosphate produced by Golgi sulfotransferases during glycosaminoglycan sulfation, and its down regulation thus may contribute to the failure of the cartilage spike to undergo osteogenesis. INPP5F is a phosphatase that preferentially hydrolyzes phosphatidylinositol 4,5 bisphosphate (PIP_2_), but also phosphatidylinositol 3,4,5 trisphosphate (IP_3_), key components of the IP_3_/DAG signaling pathway. It also modulates the AKT/GSK3B pathway by decreasing the phosphorylation of these molecules. Down regulation of this enzyme thus would lead to degradation of β-catenin and disruption of Wnt signaling.

***Phospholipase C (1):*** This enzyme, which cleaves PIP_2_ to the important second messengers IP_3_ and DAG, was up regulated at 12 dpa with FC of 2.12.

***Calcium Binding and Translocation Proteins (7):*** Five K+ and Zn^2+^ ion binding proteins were highly up or down regulated, but the focus here is on calcium binding proteins, because calcium released into the cytosol from the endoplasmic reticulum by IP_3_ is an important second messenger in intercellular signaling.

Three Ca^2+^ transport proteins, ATP2A1 (12 dpa), ATP2A3 (12 dpa) and PVALB (parvalbumin) (12 dpa) were down regulated. ATP2A1 and ATP2A3 both catalyze the hydrolysis of ATP coupled with translocation of cytosolic calcium to the lumen of the sarcoplasmic reticulum, suggesting that down regulation in muscle would increase Ca^2+^ concentration in the cytosol and therefore availability for translocation of PKC to the cell membrane. PVALB (parvalbumin, 12 dpa) is structurally similar to calmodulin and troponin C, and is thought to be involved in muscle relaxation. This protein had a FC of -5.56.

Four Ca^2+^-dependent phospholipid binding proteins were up regulated: ANXA1 (12 dpa), ANA2-A (12 dpa), ANXA8 (12 dpa), and S100A10A (12 dpa). ANXA1 promotes membrane fusion and is involved in exocytosis. It regulates phospholipase A2 activity and may be anti-inflammatory. ANXA2-A is a membrane binding protein that functions as an autocrine factor to increase osteoclast formation and bone resorption, which is important for urodele blastema formation, and may also be involved in heat-stress response. S100A induces dimerization of the ANXA2 monomer. ANXA8 is an anticoagulant protein that directly inhibits the thromboplastin-specific complex. ANXA5 was up regulated with FC <2.

***Wnt Pathway (2):*** Calveolin 1 (1, 12 dpa) is an up regulated integral membrane scaffolding protein that regulates G-protein activity and plays a role in integrin signaling. It also recruits beta catenin to calveolar membranes and thus may regulate intracellular signaling b-catenin in the Wnt pathway. On the other hand, BRD7 (12 dpa), which activates the Wnt pathway in a DVL1-dependent way by inducing phosphorylation of GSK3B, was down regulated.

***Notch Pathway (1):*** DTX1 (protein Deltex 1, 12 dpa), which acts as a positive regulator of the Notch-signaling pathway, was up regulated.

***Other Signals (4):*** RUFY3, chordin, and STAR were up regulated. RUFY3 (RUN and FYVE domain) (7, 12 dpa) is active in filopodia and growth cones and has been implicated in single axon formation by developing neurons, so may be important for axon elongation in the fibroblastema. Chordin (5, 7, 12 dpa), an antagonist of BMP, is known to dorsalize early vertebrate embryonic tissues by binding to ventralizing BMPs and sequestering them in complexes so they cannot interact with their receptors. STAR (steroidogenic acute regulatory protein, 5, 7, 12 dpa), plays a key role in steroid hormone synthesis.

WWC1 (5, 7, 12 dpa) was down regulated. It is a probable regulator of the Hippo/SWH (Sav/Wts/Hpo) signaling pathway, which plays a pivotal role in tumor suppression by restricting proliferation and promoting apoptosis.

**2. Intracellular Transport (29)**: Much of the intracellular transport of proteins, including signaling proteins, from the ER through the trans Golgi network to the cell surface and endosomes is done by sequestering the proteins in coated vesicles. For their assembly and docking to other membranes, these vesicles require small GTPases and other proteins, including the highly conserved ARF and SAR proteins that control assembly of different vesicle coats, and proteins of the RAB GTPase family.

***Vesicle Associated Small GTPases (5):*** Only one vesicle-associated GTPase, Rab 11.B1 (12 dpa) showed high positive FC. This GTPase is associated with recycling endosomes. In polarized epithelial cells, Rab 11b has been shown to localize and regulate the apical recycling compartment.

Four vesicle-associated GTPases had negative FC. ICA1 (5, 7, 12, dpa), islet cell autoantigen 1, is thought to be a target in autoimmune diabetes and may play a role in forming secretory neurotransmitter vesicles. The FC for ICA1 plunged to -11.7 at 12 dpa. RAB3GAP1 (1, 5, 7, 12 dpa) regulates the exocytosis of neurotransmitters and hormones. TBC1D7 (1, 5, 7, 12 dpa) and TBC1D17 (12 dpa) are RAB GTPase activators. The FCs of RAB3GAP1 and TBC1D7 are -8.0 and -8.7, respectively at 12 dpa.

***Other Vesicle Associated Proteins (13):*** Five of these were up regulated. NAPA (12 dpa) is required for vesicular transport between the endoplasmic reticulum and the Golgi apparatus. It is a member of the soluble NSF attachment protein (SNAP) family. SNAP proteins play a critical role in the docking and fusion of vesicles to target membranes as part of the 20S NSF-SNAP-SNARE complex. HGS (12 dpa) is a hepatocyte growth factor-regulated RTK that regulates endosomal protein sorting and plays a critical role in the recycling and degradation of membrane receptors. HGS sorts monoubiquitinated membrane proteins into the multivesicular body, targeting these proteins for lysosome-dependent degradation. When associated with STAM2, it down regulates RTK activity. STAM 2 (12 dpa) has a protein sorting function when complexed with HGS. SNX7 (5, 7, 12 dpa) is also involved in vesicular protein sorting. VPS28 (1, 5, 7, 12 dpa) facilitates the cycling of proteins through the trans-Golgi network and plays an important role in segregating proteins into the appropriate organelles such as endosomes, lysosmes, and plasma membrane. VSP 28 and SNX7 had FCs of 5.97 and 7.3, respectively, at 12 dpa.

Eight proteins of this group were down regulated. USP33 (12 dpa) is a deubiquitinating enzyme that plays a role in axon guidance and acts as a regulator of G-protein coupled receptor signaling and plays a central role in recycling of beta 2 adrenergic receptors. CD36L2 (12 dpa) participates in membrane transportation and reorganization of endosomal/lysosomal compartments. AP1G2 (5, 12 dpa) is an adaptin protein that promotes formation of clathrin-coated pits and vesicles for transport of ligand-receptor complexes from the plasma membrane or from the trans-Golgi complex to lysosomes. It is strongly down regulated at 12 dpa. SCFD1 (5, 12 dpa) is involved in vesicular transport between the ER and Golgi and in vesicle docking during exocytosis. CHMP4A (7, 12 dpa) and CHMP2B (5, 7, 12 dpa) are components of the endosomal ESCRT-III complex that degrades, sorts and recycles surface receptors. SCRN1 (7, 12 dpa) regulates exocytosis in mast cells. MYO6 (5, 7, 12 dpa) is a reverse direction motor protein involved in vesicle trafficking. Myo6, CHMP4A, and CHMP2B are heavily down regulated at 12 dpa, with FC of -4.5, -9.7, and -14.9, respectively.

***Non-Vesicular Transport (10):*** Non-vesicular transport proteins are proteins that anchor other proteins to membranes, import and export nuclear proteins, transport nucleosides and neural mRNA, and facilitate nuclear pore assembly. Four such proteins were up regulated. GOSR-1 (5 dpa) facilitates transport from the Golgi to the plasma membrane. GKAP1 (12 dpa) anchors protein kinase 1 alpha to Golgi membranes. NTF2 (12 dpa) is a cytosolic factor that facilitates protein transport into the nucleus. CSE1L (5, 7, 12 dpa) acts as a receptor to facilitate transport of importin-alpha into the nucleus.

Six proteins were down regulated. SAR1a (5, 7, 12 dpa) is a GTPase involved in transport from the ER to the Golgi. NUP93 (5, 7, 12 dpa) is required for correct nuclear pore assembly. SLC28A2 (5, 7, 12 dpa) is essential for nucleoside transport and STAU2-A (5, 7, 12 dpa) is a RNA binding protein required for microtubule-dependent transport of neuronal mRNA from cell body to dendrite for protein synthesis. PLEKHA1 (12 dpa) binds specifically to phosphatidylinositol-3, 4-diphosphate to recruit it to the plasma membrane. VPS45 (12 dpa) is involved with protein trafficking through the trans-Golgi network and vesicle docking during exocytosis. XPO4 (1, 5, 7, 12 dpa) mediates the transport of proteins between the nuclear and cytoplasmic compartments. SCLC28A, STAUA, and NUP93 are exceptionally down regulated at 12 dpa, with FCs of -5.94, -10.76, and -12.64, respectively.

***Extracellular Transport (1):*** Most of the proteins in this category were hemoglobin subunits. Only one, HBB1 (1, 5, 12 dpa), was up regulated by a FC >2; none were down regulated.

**3. Transcription**

***Chromatin Associated Proteins (9):*** Chromatin associated proteins were primarily histones or histone binding proteins involved in regulating the state of chromatin condensation and remodeling (alteration of DNA-nucleosome topology). Four proteins were up regulated. BTBD17 (12 dpa) may function as a transcriptional repressor, but its *in* vivo function has not been defined. HIST1H1D (12 dpa) is a linker histone essential for chromatin condensation. The high mobility group proteins HMGB2 (12 dpa) and HMGX (12 dpa) are non-histone DNA binding proteins that facilitate cooperative interactions between cis-acting proteins.

Five proteins were down regulated. ACTL6A (12 dpa) is a component of a HAT complex that activates transcription by acetylation of histones H4 and H2A. NCOR1 (1, 12 dpa) mediates ligand independent transcriptional repression of thyroid hormone and RA receptors by promoting histone deacetylation and chromatin condensation. It was down regulated by over a factor of 4 at 12 dpa. SIN3B (1, 5 dpa) represses transcription by serving as a scaffold to tether HDAC enzymes and thus prevent histone deacetylation. LRB (7, 12 dpa) attaches chromatin to nuclear envelope and helps maintain chromatin structure. POLR1A (1, 5, 7, 12 dpa) is the large subunit of a DNA-dependent RNA polymerase; it had a FC of -5.3 at 1 dpa and -14.36 at 12 dpa.

***Transcription Factors (25):*** Twelve transcription factors were up regulated. PIAS4, MEOX2, LRRFIP1, and TAF3 were up regulated only at 12 dpa. PIAS4 (12 dpa) functions as an ezrin-type small ubiquitin-like modifier (SUMO) ligase. It is a negative regulator of transcription in the Wnt, STAT, p53 and steroid hormone pathways. MEOX2 may play a role in the regulation of limb myogenesis. Since the Xenopus regenerate does not possess muscle, it may regulate muscle formation in the stump region just proximal to the fibroblastema. MEOX2 is a target gene of the TGF-β pathway that regulates the development of skeletal, cardiac and smooth muscle. LRRFIP1 is a transcriptional repressor for genes encoding TNF, EGFR, and PDGFA, and controls smooth muscle proliferation by repressing PDGFA after injury. TAF3 (TFIID) is one of the general transcription factors required for transcriptional initiation by RNA polymerase II.

SIN3B (1, 5 dpa) is a transcriptional repressor. RUNX1T1 (1, 12 dpa) is a member of the myeloid translocation gene family that regulates transcription by binding to histone deacetylases and transcription factors. HNF1B (7, 12 dpa) is a hepatic transcription factor and SNIP1 (7, 12 dpa) is a SMAD nuclear interacting protein. Increased SNIP1 expression inhibits BMP-induced gene responses. BMP signaling is important for a wide array of developmental processes.

REST-A, RNF168, and ZHX3 were all up regulated at 5, 7, and 12 dpa, and FOXD5 and D5-B were up regulated at 1, 5, 7, and 12 dpa. REST A represses transcription of neuronal genes in non-neural tissues. RNF168 is an E3 ubiquitin ligase required for accumulation of DNA repair proteins at sites of DNA damage. ZHX3 is a zinc finger transcriptional repressor. FOXD5 and FOXD5-B play a role in axon extension and in cartilage development. FOXD5, FOXD5-B, SNIP1, and ZHX3 showed exceptional up regulation at 12 dpa, with FCs of 4.73, 4.79, 6.8, and 14.13, respectively. Two proteins known to be involved in limb development and regeneration, SALL1 and LHX9, were part of our data. SALL1 is a zinc finger transcriptional repressor that was up regulated at a FC <2. LHX9 is a homeodomain protein that was down regulated at <2 FC. LHX9, along with LHX1 and 2, integrates signaling events that link limb patterning and outgrowth in all three axes.

Thirteen transcription factors were down regulated. SSB and NF7 were down regulated with FC > 2 only at 1 dpa. SSB is a transcription termination factor. Nuclear factor 7 is a putative maternal transcription factor selectively retained in the cytoplasm from fertilization to mid-blastula in Xenopus and functions in DV patterning. PTF1A is a transcriptional activator that was down regulated only at 7 dpa. PIAS1-A, ANKRD1, CNOT10, E2F8, and PAX 1 were down regulated only at 12 dpa. ANKRD1 is a transcription factor localized specifically in the nucleus of endothelial cells and is induced by IL-1 and TNF-α stimulation. CNOT10 is a subunit of the CCR4-Not transcriptional regulation complex that is also an effector of mRNA decay. E2F8 directly represses a subset of E2F1-dependent transcription of cell cycle progression genes. PAX1 is a transcriptional activator belonging to the PAX family of proteins that interacts with MEOX2. It plays an important role in vertebrate embryonic pattern formation.

DIP2C, GCM1-A, NR4A1, and SIX4.2 all were down regulated at 5, 7, and 12 dpa. DIP2C shares similarity to a Drosophila protein that interacts with the *disconnect* patterning gene in the nervous system. GCM1-A is a homolog of the Drosophila glial cells missing transcription factor, and NR4A1 is a member of the steroid-thyroid hormone-retinoid superfamily that induces apoptosis when translocated from the nucleus to mitochondria. SIX4.2 may have a role in otic, olfactory, and optic neural cell differentiation. PATZ1 is a transcriptional repressor that was down regulated at all dpa. DIP2C, PATZ1, and GMC1-A showed exceptionally strong FC of -5.5, -8.5, and -13.65, respectively at 12 dpa.

***RNA Processing Proteins (12):*** Eight proteins were up regulated. BXDC5, HNRNPH1, LOC494754, and LSM14A were up regulated at 12 dpa. BXDC5 has been implicated in ribosome biogenesis. HNRNPH1 and LOC494754 are proteins that bind to heterogeneous nuclear RNA and are involved in RNA splicing and metabolism. LSM14A functions in the formation of P-bodies, cytoplasmic structures that provide storage sites for non-translating mRNAs. Several dead box (DDX) proteins were detected. These proteins contribute to the regulation of differentiation by their ability to modulate RNA secondary RNA structure, and thus availability, in processes such as ribosome and spliceosome assembly, translation initiation and RNA splicing. DDX18 and DDX54 were up regulated only at 12 dpa. DDX18 plays an important role in cell proliferation and DDX54 represses the transcriptional activity of nuclear repressors. DDX21 was up regulated at 1 and 12 dpa. This is a nucleolar protein involved in the processing of 20s rRNA to 18s rRNA. LRPPRC (7, 12 dpa) plays a role in RNA metabolism in both nucleus and mitochondrion.

Four processing proteins were down regulated. U2AF1 is down regulated at 5 and 12 dpa, and CCNL2 (cyclin 2) at 7 and 12 dpa; both are splicing factors. CCNL2 is a novel member of the cyclin family. Overexpression inhibits proliferation and differentiation of mouse EC p19 cells and induces them to undergo apoptosis. RNPS1-A and SCNM1 are down regulated at 5, 7, and 12 dpa. SCNM1 functions as a splicing factor; RNPS1-A is part of a post-splicing multiprotein complex that detects exported mRNAs with truncated open reading frames and initiates nonsense-mediated mRNA decay. The FC at 12 dpa for RNPS1-A, CCNL2 and SCNM1 was high, -5.76, -6.2, and -10.18, respectively.

**4. Translation (9):** A large number of ribosomal proteins appeared in our data. None of the down regulated and only three of the up regulated ribosomal proteins showed FC >2 on one or more dpa. The latter were the 60s RPL10A and RPL35A proteins, which were up regulated at 5, 7 and 12 dpa, and the 60s protein MGC114621, which was up regulated 7 and 12 dpa.

Translation factors that appeared included initiation factors (eif), elongation factors (eef), amino acyl tRNA synthetases, and tRNA ligases. Four tRNA-related proteins were up regulated, LRRC47 and SEPSECS at 1 dpa, LARS at 12 dpa, and IARS at 7and 12 dpa. LRRC47, LARS, and IARS function as tRNA ligases. SEPSECS is a tRNA synthase essential for the synthesis of proteins that contain selenium. Two elongation factor isoforms (EEF1A1 and EEF1A2) that were up regulated with FC <2 at 5, 7, and 12 dpa were nevertheless of interest. EEF1A1 is a developmental isoform that is greatly increased relative to EEF1A2 after denervation of regenerating rat muscle. EEF1A2 also interacts with PRDX-1 to provide cells with extraordinary resistance to oxidative stress-induced apoptosis.

One initiation and one elongation factor were down regulated. EIF3C was down regulated at 12 dpa and DPH5 at 1 and 12 dpa. DPH5 is involved in the synthesis of dipthamide, a highly conserved, post-translationally modified histidine residue found only on translation elongation factor 2. EIF3C is a component of the eukaryotic translation initiation factor 3 complex that associates with the 40s ribosomal subunit to recruit other elongation factors of the 43s pre-initiation complex. It also interacts with the neurofibromin (NF2) cytoskeletal protein.

**5. Cytoskeleton:**

***Muscle Proteins (16):*** MYBPC3, the cardiac isoform of myosin-binding protein C found in the cross-bridge-bearing zone (C region) of striated muscle A bands, was the only protein up regulated by 2X or more, at 7 and 12 dpa. Muscle proteins with negative FC >2 at 12 dpa were ACTA1 (apha actin 1), DAG1 (dystrophin-associated glycoprotein), MYH 2, MYH 8, MYH 13, MYLPF, SMPX (small muscle protein), TPM1, TPM2, and TMOD4. ACTN3 (alpha 3 actinin), MHC alpha, MYL1 and TMOD4 were down regulated at 7 and 12 dpa. CNN3 was down regulated at 5, 7, and 12 dpa, where it reached negative 8.43. CNN3 is an actin binding protein that plays a role in actomyosin structural organization; this level of down regulation suggests considerable disorganization of stump muscle, consistent with histological observations.

***Non-Muscle Proteins (34):*** Many of these proteins bind actin and ATP to regulate organization of the cytoskeleton, others are intermediate filaments (primarily keratins), while still others are involved in adhesion. The non-muscle proteins showed a much higher level of up regulation than down regulation. Twenty-five proteins showed up regulation and 7 were down regulated.

Thirteen intermediate filaments were up regulated. These were the epithelial keratins KRT13, KRT19, KRT5.2, KRT6, XAK-a, XAK-c, KRT 14, keratin-3, type I cytoskeletal 51 kDa, all at 12 dpa; KRT15 (7, 12 dpa), keratin type II cytoskeleton 1 (7, 12 dpa), and two other intermediate filaments, vimentin (VIM1) (7, 12 dpa) and MGC84118 (12 dpa). KRT14 was the most highly up regulated intermediate filament at 4.52.

Up regulated proteins involved in actin binding or polymerization were CDC42EP4, CORO1a, SHROOM2, myl12b, and LOC443650, all at 12 dpa. CDC42EP4 is a Ras GTPase belonging to the p21 Rho family that regulates actin localization, nuclear displacement and protein trafficking; CORO1A is crucial to the formation of lamellapodia and filopodia in highly motile cells; SHROOM interacts with actin filaments and may be involved in endothelial cell morphology. KLC4 (12), PAFAHB1-B (5, 7, 12), and MYO1D (1, 5, 7, 12) function in microtubule organization. PAFAHB1-B was the most highly up regulated of these, 6.48 at 12dpa. This protein enhances dynein-mediated microtubule sliding by targeting dynein to the microtubule plus end. It may play a role in migration of fibroblasts during wound healing and by extension, in fibroblastema formation.

Other up regulated proteins involved in functional or structural (cell junctions) adhesion were desmoplakin (DSP) (12 dpa), integrin alpha chain V (ITGAV) (12 dpa), NF2 (12 dpa), DSCAM (1, 5 dpa) and integrin beta 1 (ITGB1) (7, 12 dpa) are involved in functional or structural (cell junctions) adhesion. NF2 (neurofibromin) is similar to the ezrin, radixin, moesin (ERM) family that is thought to link cytoskeletal components to cell membrane proteins. Ezrin was also up regulated in our data, but by much less than 2.

The down regulated non-muscle proteins were EML1 (7, 12 dpa) and INTU (12 dpa), both involved in microtubule assembly and organization; the ATP and actin binding proteins FHOD1 (12 dpa), STK35 (5,7, 12 dpa), TMSB4X (12 dpa) and GAP 43 (5, 7, 12 dpa); and TLN (12 dpa) and EPB4 (7 dpa). FHOD1 is required for assembly of F-actin structures such as stress fibers and plays a role in cell elongation by coordinating the organization of actin fibers and microtubules. Talin and EBP4 are adhesion proteins. Talin plays a significant role in assembling actin filaments with integrins to link the cell membrane to ECM constituents. TMSB4X (thymosin β4) binds to actin monomers to prevent their polymerization, and is thus involved in cell proliferation, migration, and differentiation. Thymosin β4 also stimulates higher production of the survival kinase PKC (Akt). GAP-43 is a nervous system-specific membrane adaptor protein that enables the association of phosphatidylinositol-4-5 bisphosphate and PIP2 with actin to facilitate actin polymerization, thereby regulating neuronal structure. It is expressed at high levels in neuronal growth cones and is considered to be a crucial component of an effective axon regenerative response.

**6. ECM (10):** Eight proteins were up regulated, of which five are involved in establishing the provisional fibrin matrix during hemostasis. These are FGA (1 dpa), FGB (1 dpa), FGG (1, 5 dpa), PROC (1, 12 dpa), and THBS2 (12 dpa). The other three up regulated proteins were ALPL (tissue non-specific alkaline phosphatase, 12 dpa), ITGA2B (1, 5, 12 dpa), and TGFBI (12 dpa). ALPL may play a role in skeletal mineralization. ITGA2B encodes the integrin alpha chain 2b, which along with β-3 integrin forms a fibronectin receptor expressed in platelets that plays a crucial role in coagulation. Because it continues to be expressed after clotting has occurred, it may also participate in cell-surface mediated signaling. TGFBI (transforming growth factor beta-induced) is an RGD-containing protein induced by TGF-β that binds to collagens I, II and IV and inhibits cell adhesion. Also of interest, but up regulated with FC < 2, were matrilin 2, a member of the von Willebrand factor A domain-containing family that is involved in matrix assembly, and fibronectin, which is involved in blood coagulation, cell adhesion, and cell migration during wound repair..

Down regulated ECM proteins were the hemostatic coagulation factor 2 (F2, 12 dpa) and MFAP1 (1, 5, 7, 12 dpa), a component of elastin-associated microfibrils.

**7. Metabolism:**

***Carbohydrate (12):*** In the TCA cycle, ATP5F1 (12 dpa) and MGC68826 (STEAP4, 5, 12 dpa) were up regulated. ATP5F1 is one of several subunits of the H+ mitochondrial ATP synthase transport complex, most of which were down regulated. MGC68826 is a hypothetical Xenopus protein that binds flavin dinucleotide and has electron carrier and oxidoreductase activities. Two proteins were down regulated, NDUFA4 (12 dpa) and NDUFA9 (7, 12 dpa). These are two of several proteins with dehydrogenase (ubiquinone) activity that are also involved in mitochondrial electron transport and fatty acid biosynthesis.

No glycolytic proteins were up regulated, but four were down regulated. ALDOA (12 dpa) plays a key role in glycolysis and glyconeogenesis. HK1A (hexokinase, 1, 5, 7 dpa) is a ubiquitous form of hexokinase that localizes to the mitochondrial outer membrane. PGAM2 (phosphoglycerate mutase, 12 dpa) interconverts 3 and 2-phosphooglycerate. PYM (12 dpa) is an important allosteric enzyme in glycolysis. Also down regulated were four enzymes involved in glycosylation. B3GNT (5, 7, 12 dpa) and FUT4 (5, 7, 12 dpa) are Golgi membrane proteins with galactosyltransferase and fucosyltransferase activity for amino acids. GAINT (12 dpa) is a glycopeptide transferase involved in O-linked oligosaccharide biosynthesis that transfers an N-acetyl-D-galactosamine residue to an already glycosylated peptide. GCNT1 (5, 7, 12 dpa) is essential for the formation of the core O-glycan branch.

***Non-Carbohydrate (19):*** These were enzymes involved in the metabolism of lipid, protein, nucleic acids, iron and vitamins. Five proteins involved in lipid metabolism were up regulated. ACADM (acyl-CoA dehydrogenase, 1, 5 dpa) and ACAA2 (1, 12 dpa) catalyze the initial and final steps of the mitochondrial fatty acid beta-oxidation pathway, respectively. Apolipoprotein A1 (1, 5 dpa) is the major protein component of high-density lipoprotein HDL and HDLBP (12 dpa) binds HDL; together they regulate cholesterol accumulation.

MGC52912 (dehydrodolichyl diphosphate) has alkyl or aryltansferase activity and was down regulated at 12 dpa.

There were six enzymes involved in protein and amino acid metabolism. SH3TC1 was very highly up regulated with FC of 4.05, 5.31, and 13.24 at 5, 7 and 12 dpa. There is no functional information on this protein, but the level and pattern of its up regulation suggests it is of some importance. The others were down regulated. CKM (12 dpa) plays a central role in skeletal muscle energy transduction. AADAT (5, 12 dpa) is a transaminase with broad substrate specificity. AGXT2L1 (12 dpa) catalyzes the pyridoxal-phosphate-dependent breakdown of ethanolamine, converting it to ammonia, inorganic phosphate and acetaldehyde. GPT2 (12 dpa) catalyzes the reversible transamination between alanine and 2-oxoglutarate to form pyruvate and glutamate.

Other up regulated metabolic enzymes were MTHFSD (methenyltetrahydrofolate synthesis domain containing, 12 dpa), ACO1 (acontinase 1, 1, 5, 7, 12 dpa), and MGC147117 (5, 7, 12 dpa). MTHFSD is involved in vitamin metabolism and ACO1 catalyzes the isomerization of citrate to isocitrate via cis-acontinate. MGC147117 is an uncharacterized protein with similarity to proteins having oxidoreductase activity. The iron transport molecule transferrin, which is essential for cell division, is up regulated on all dpa, but with FC well below 2.

Down regulated enzymes were RBM12B (7, 12 dpa), FECH (ferrochelatase, 7, 12 dpa), RDHL (12 dpa), LOC495283 (1, 5 dpa) and MGC147239 (12 dpa). LOC495283 is an uncharacterized protein similar to 5-aminolevulinate synthase and MGC147239 is an uncharacterized protein that has hydrolase activity. RBM12B is an RNA binding protein and RDHL is an oxidoreductase involved in retinoic acid biosynthesis.

**8. Cell Protection:** These proteins fit into three subcategories, inflammatory-related, apoptosis-related, and chaperones, each of which had several proteins that were up or down regulated by a factor of 2X or more on one or more dpa.

***Inflammatory-related (12)***: These proteins are involved in defense against bacteria and toxins and in drug metabolism. There were five up regulated proteins, TM9S4 (1, 5, 7, 12 dpa), XENOXIN 1 (5, 12 dpa), NR1L2 (PXR, 1, 5, 7, 12 dpa), CSF3R (5, 7, 12 dpa) and LYSMD1 (7, 12 dpa). TM9S4 belongs to the nonaspanin family, whose members are thought to function in internalization of bacteria by phagocytes. XENOXIN-1 belongs to a family of peptides related to snake venom cytoxins and neurotoxins and is a defense peptide expressed by skin dorsal glands in Xenopus. NR1L2 is a nuclear receptor that serves as a generalized sensor of hydrophobic toxins. CSF3R plays a crucial role in neutrophil production. LYSMD1 is a putative peptidoglycan binding protein involved in cell wall metabolic processes, but its function is unknown.

Seven proteins were down regulated. CYP1A1 (1, 5, 7, 12 dpa), CYP2C8 (5, 7, 12 dpa), and CYP3A4 (12 dpa) are members of the cytochrome 450 family involved in drug metabolism. MMP6 (MMP25) is a member of the membrane-type MMP (MT-MMP) subfamily, attached to the plasma membrane via a glycosylphosphatidyl inositol anchor. In response to bacterial infection or inflammation, it is thought to inactivate α-1 proteinase inhibitor, a major tissue protectant against proteolytic enzymes released by activated neutrophils, facilitating the transendothelial migration of neutrophils to inflammatory sites. FMO5 (1,5,7,12 dpa) is a flavin containing monooxygenase involved in drug metabolism. PGLYP1 (1 dpa) has bactericidal activity toward Gram-positive bacteria and bacteriostatic activity toward Gram-negative bacteria. TEgg023j21.1-001 (7, 12 dpa) is a cytosolic glutathione S-transferase isozyme that catalyzes conjugation of the reduced form of glutathione to xenobiotic substrates for the purpose of detoxification. CYP2C8, FMO5 and CYP1A1 showed negative FCs of 4.43, 4.50, and 15.7, respectively.

***Apoptosis- related (6):*** Our data contained about equal numbers of pro and anti-apoptotic proteins, but those which showed the highest FC are primarily pro-apoptotic, and nearly all of these were up regulated. MGC83934 (PARP, 9, 5, 7, 12 dpa) is a poly(ADP-ribose) polymerase activated by DNA damage. The FC for PARP is 21.7 at 12 dpa, among the highest encountered in our data. TGM2 (1, 5, 7, 12 dpa) is a pro-apoptotic, calcium-dependent transglutaminase that cross-links proteins and conjugates polyamines to proteins. TM2D3 (7, 12 dpa) has a regulatory role in apoptosis. TRAF4 (12 dpa) is a pro-apoptotic member of the TNF receptor associated factor (TRAF) family that links the TNFR family to different signaling pathways.

Two down regulated pro-apoptotic proteins were BAX-A (12 dpa), a member of the BCL2 family that functions to antagonize the apoptosis repressor BCL2 and induce release of cytochrome C, leading to the activation of caspase 3. The FC for BAX-A was -9.0. TIA1 (12 dpa) is a member of a RNA-binding family that induces apoptosis in cytotoxic lymphocyte cells.

***Chaperones (3)***: Two chaperones were up regulated, HSP110 (12 dpa) and PDIA4 (12 dpa). HSP110 is an ATP binding heat shock protein that prevents aggregation of denatured proteins in cells under severe stress. PDIA4 is one of several disulfide isomerases in the endoplasmic reticulum that play a role in the folding of disulfide-bonded proteins.

DNAJC9 (5, 7, 12) had the highest down regulation of any protein in our data, negative 32.8 at12 dpa and well over negative 2.0 at 5 and 7 dpa. It is a J protein that acts as a co-chaperone with HSP70. J proteins function in many cellular processes by regulating the ATPase activity of 70kDA heat shock proteins. DNAJC9 is induced by ER sress and plays a role in protecting stressed mammalian cells from apoptosis.

**9. Degradation:**  These proteins could be subdivided into those associated with ubiquitination and the proteasome, and protease degradation of carbohydrates, lipids, protein, and nucleic acid.

***Ubiquitin/ Proteasome Associated (6) :*** Three proteins were up regulated at 12 dpa. MTBP plays a role in MDM2-dependent TP53/p53 homeostasis in unstressed cells by promoting MDM2-mediated ubiquitination and subsequent degradation of TP53/p53. PSMA6 and PSMB6, along with several other PSM proteins that were up regulated with FC < 2, are proteasome subunits.

Three proteins were down regulated***.*** PSMD12 (5, 7, 12) is a proteasome subunit and UBR2 (12 dpa) is a ubiquitin E3 ligase that targets proteins for degradation. RNF216 (12 dpa) acts as an E3 ubiquitin ligase that accepts ubiquitin from specific E2 ubiquitin-conjugating enzymes and then transfers it to substrates promoting their degradation by the proteasome.

***Other (6):*** Four proteins were up regulated. PSAP (12 dpa) is a glycoprotein precursor for saponins A-D which localize to the lysosomal compartment where they facilitate catabolism of glycosphingolipids with short oligosaccharide groups. It stimulates neurite outgrowth and enhances nerve regeneration. Cathepsin B (12 dpa) is a lysosomal cysteine protease that degrades beta-amyloid and other proteins and activates enzymes. DCP2 (12 dpa) is a de-capping complex required for removal of the 5-prime cap during mRNA degradation. OVCH1 (ovochymase, 1, 5 dpa) is a chymotrypsin-like serine protease released during Xenopus egg activation and apparently maintained in metamorphosed frogs.

There were two proteins down regulated at 12 dpa. Darmin is a caroxypeptidase that is a marker for early endoderm cells in Xenopus. DPYS is involved in the degradation of nucleic acid pyrimidines. Another protein of possible interest that is down regulated with FC < 2 is PNPLA4, a hydrolase for retinyl esters in keratinocytes. Retinyl esters serve as a source for retinoic acid, and their down regulation might lead to RA deficiency.

**10. Cell Cycle:** These proteins could be separated into proteins for specific cell cycle phases and others.

***G1/S (8):*** Three proteins were up regulated at 12 dpa. CDC25-1-A activates (dephosphorylates) cyclin-dependent kinase CDC2 and is required for progression from G1 to S. It is specifically degraded in response to DNA damage, preventing cells with chromosomal abnormalities from progressing through division. RNSEH2A degrades the RNA of RNA:DNA hybrids. It participates in DNA replication by mediating the removal of lagging strand Okazaki fragment RNA primers. TBRG1 causes G1 arrest and can inhibit DNA synthesis.

Five proteins were down regulated. LIG1 (12) is a DNA ligase that seals nicks in double-stranded DNA during DNA replication and repair. SMC6 (5, 12 dpa) is also involved in DNA repair. NBN (1, 5, 7, 12) plays a critical role in the cellular response to DNA damage and the maintenance of chromosome integrity. It plays a major role in control of the Rb checkpoint during G1 and may be involved in the G2 checkpoint as well. RAD52 (1, 5, 7, 12 dpa) and ZMCM6A (12 dpa) are essential for the initiation of DNA replication. They act as components of the MCM complex, which is the putative helicase for DNA replication.

***G2/M Phase (11)***: Seven proteins were up regulated. ANlN (5, 12 dpa) is an anillin actin binding protein, essential for cytokinesis and structural integrity of the cleavage furrow and completion of furrow ingression. NUMA1 (5, 7 dpa) is a mitotic apparatus component that is required for the formation and maintenance of mitotic spindles. SASS6 (7, 12 dpa) is required for centrosome duplication and is strongly up regulated at 12 dpa with FC of 5.77. STAG2 (5, 7 dpa) is a component of the cohesion complex, which is required for cohesion of sister chromatids after DNA replication. TP53BP2 (tumor protein p53 binding protein 2, isoform 1) (5, 7, 12 dpa) is a negative regulator of the cell cycle that impedes cell cycle progression at G2/M and also enhances DNA binding and the transactivation function of TP53 on the promoters of pro-apoptotic genes. It is strongly up regulated, with FC of 4.1, 5.2, and 14. PAFAH1B-1B (5, 7, 12 dpa), is involved with establishing mitotic spindle orientation; its FC at 12 dpa was 6.48. RECQL4 (12 dpa) has helicase activity and is involved in recombination.

Four proteins were down regulated. CHEK1 (5, 7, 12 dpa) is required to mediate G2/M cell cycle arres in response to DNA damage or the presence of unreplicated DNA, and was strongly down regulated at 12 dpa to -4.55. NEK6 (1, 5, 7, 12 dpa) is required for chromosome segregation at the metaphase-anaphase transition and thus for mitotic progression, and reached a FC of 9.2 by 12 dpa. TACC3 (5, 7, 12 dpa) is a motor spindle protein that may play a role in the stabilization of the mitotic spindle. Rac GTPase activating protein 1 (5, 7, 12 dpa) is part of the centralspinlin complex that serves to signal myosin contractile ring formation during cytokinesis.

***Other (5)***: Four proteins were up regulated. ECD (protein ecdysoneless homolog, 1, 5, 7, 12 dpa) is a novel regulator of p53 stability and function and may also be a transcriptional regulator for the expression of glycolytic genes. SUMO2 (12 dpa) is a ubiquitin-like protein that is not involved in protein degradation by the proteasome and may function as an antagonist of ubiquitin in the degradation process. It effects post-translational modification on lysine residues of proteins that play a crucial role in cellular processes such as nuclear transport, DNA replication and repair, and mitosis. UBE2n (12 dpa) is a E2 ubiquitin-conjugating enzyme that also is not involved in proteasomal degradation and plays a role in post-replication DNA repair. WDHD1 (12 dpa) functions as a DNA replication initiation factor.

The single down regulated protein was UHRF1BP (12 dpa), a ubiquitin-like protein of unknown function that may act as a negative regulator of cell growth.
